# Single-cell transcriptomic atlas of the human fetal uveal tract reveals heterogeneity in melanocyte populations

**DOI:** 10.1016/j.isci.2026.117510

**Published:** 2026-09-12

**Authors:** James Draper, Ian R. Reekie, Lakshanie Wickramasinghe, Srilakshmi Sharma, Mark Coles, Andrew D. Dick, Christopher Buckley, Helen Kalirai, Sarah E. Coupland

**Affiliations:** 1 Department of Eye and Vision Science, Institute of Life Course and Medical Science, University of Liverpool, Liverpool, UK; 2 Kennedy Institute of Rheumatology, University of Oxford, Oxford, UK; 3 Institute of Ophthalmology, University College London, London, UK; 4 School of Cellular and Molecular Medicine, University of Bristol, Bristol, UK; 5 NIHR Biomedical Research Centre, Moorfields Eye Hospital, London, UK

**Keywords:** single-cell RNA sequencing, uveal tract, melanocyte heterogeneity, human fetal development, uveal melanoma, neural crest, cell-cell communication, choroid, transcriptomics, ocular development

## Abstract

Uveal melanocytes are neural crest-derived cells contributing to ocular pigmentation, yet their developmental heterogeneity and microenvironmental roles remain poorly characterized. We present a single-cell transcriptomic atlas of the human fetal uveal tract, profiling 81,603 cells across 14 cell types from eight eyes at 12 and 20 post-conception weeks. Among 5,142 melanocytes, we identify four subpopulations, including a transcriptionally unique cluster enriched in posterior uveal tissue at later developmental stages. This population expresses *NR2F1*, *FOXC1*, and *PITX2* alongside extracellular matrix and angiogenic genes and is predicted to communicate with Schwann cells, endothelial cells, and immune populations to modulate the uveal microenvironment. Multiplex immunofluorescence confirms *MITF*^+^/*FOXC1*^+^ co-expressing cells in fetal choroidal tissue, validating this population *in situ*. A refined gene signature from this cluster associates with high-metastatic risk across three independent uveal melanoma cohorts and is enriched in aggressive tumor cell states in single-cell data, implicating developmental program reactivation in disease progression.

## Highlights

• Single-cell atlas of 81,603 cells reveals 14 fetal uveal tract cell types
• Fetal uveal melanocytes comprise four transcriptionally distinct subpopulations
• Cluster 2 melanocytes show mesenchymal, vascular, and immune gene programs
• Cluster 2 signature predicts metastatic risk across three uveal melanoma cohorts

## Graphical abstract

**Figure:**
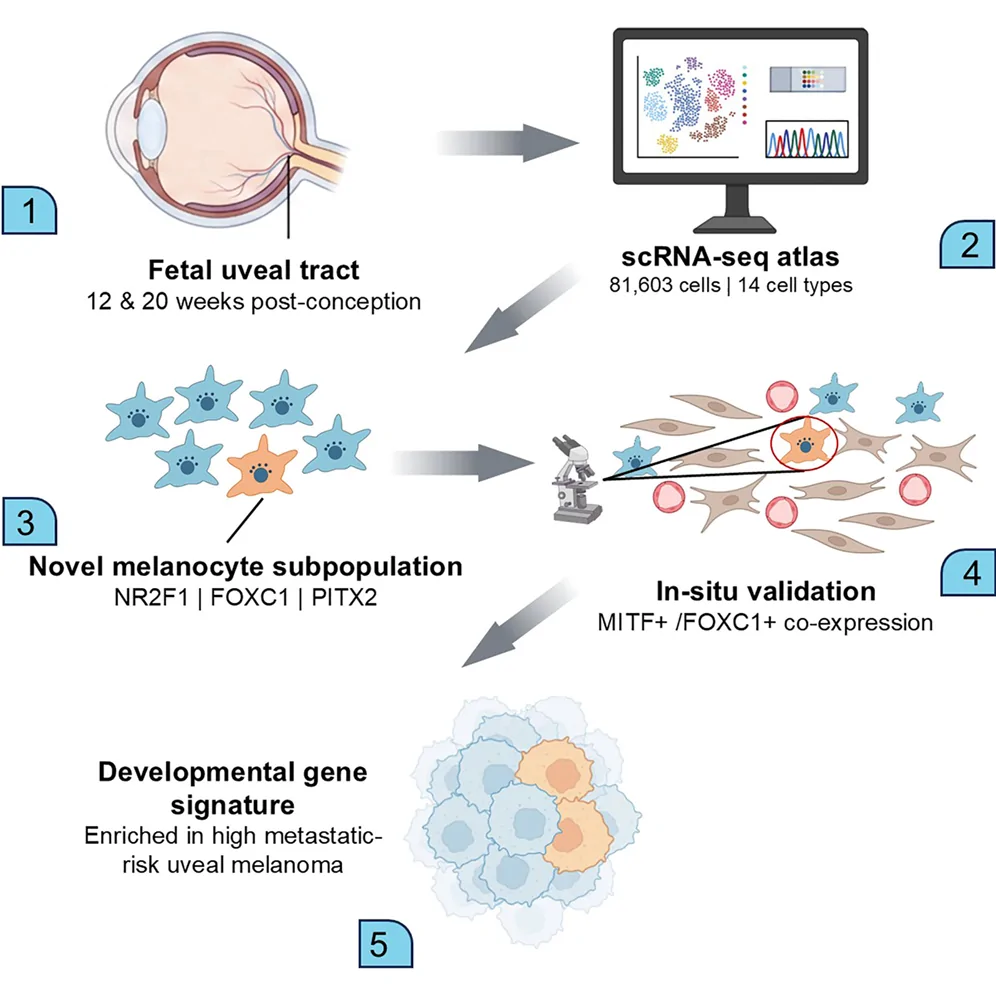

## Teaser

Cancer; Cell biology; Transcriptomics

## Introduction

Melanocytes are pigment-producing cells derived from the embryonic neural crest that migrate and differentiate to populate diverse anatomical sites, including the epidermis, hair follicles, cochlea of the ear, and uveal tract of the eye. While their role as pigment-producing cells is well established, their functions within the uveal tract, which comprises the iris, ciliary body, and choroid, remain incompletely understood. Emerging evidence suggests that uveal melanocytes contribute to photoprotection, immune regulation, and tissue homeostasis.^1^^,^^2^^,^^3^^,^^4^ The choroid is noteworthy for its unique dense vascular architecture, supplying approximately 90% of the oxygen required by the outer retina, one of the most metabolically active tissues in the human body.^4^^,^^5^^,^^6^ Some evidence suggests that choroidal melanocytes are essential for the proper development and maintenance of choroidal vasculature, though the relationship is not well defined.^7^^,^^8^

During embryogenesis, ocular melanocytes originate from the neural crest and migrate to the periocular mesenchyme, eventually populating the stroma of the emergent uveal tissues.^9^^,^^10^^,^^11^^,^^12^^,^^13^ However, the spatiotemporal dynamics of this migration, as well as the intrinsic and extrinsic factors guiding melanocyte differentiation, remain poorly characterized. Uveal melanoma (UM), which arises from uveal melanocytes, is the most common primary intraocular malignancy in adults, with 90% of cases originating in the choroid.^14^^,^^15^ Previous studies suggest that high-metastatic-risk UM exhibits transcriptomic signatures reminiscent of developmental programs,^16^^,^^17^^,^^18^^,^^19^ a phenomenon also observed in other neural crest-derived cancers.^20^ This raises the possibility that pathways active during melanocyte development may be reactivated in aggressive UM phenotypes, promoting plasticity, and dissemination.

Transcriptomic profiling has advanced the understanding of UM biology. Integrated genomic analysis of the TCGA-UVM cohort of bulk RNA sequencing (RNA-seq) data allowed subdivision of tumors into distinct molecular subsets stratified by metastatic risk.^21^ Single-cell RNA sequencing (scRNA-seq) has revealed intratumoral heterogeneity at unprecedented resolution. Pandiani et al. identified distinct cell states within primary UM tumors.^22^ Durante et al. characterized the UM microenvironment across primary and metastatic tumors, characterizing diverse immune infiltrate profiles.^23^ More recently, Li et al. characterized intratumoral malignant cell heterogeneity across 17 UM tumors, identifying distinct transcriptional subtypes with differential prognosis and immune microenvironments, deriving a prognostic gene signature validated across multicenter cohorts.^24^ Together, these studies characterized the transcriptional landscape of UM; however, the transcriptional landscape of normal uveal melanocytes during the developmental window remains uncharacterized at single-cell resolution. A fetal uveal single-cell atlas, therefore, provides a developmental reference against which the aberrant transcriptional states observed in UM can be contextualized.

To explore this hypothesis, we present a scRNA-seq analysis of the human fetal uveal tract, with a focus on melanocyte populations. By characterizing transcriptional profiles during melanocyte development and maturation, we aim to identify distinct cell states and subpopulations that may contribute to tissue formation and function. Understanding cellular heterogeneity in the fetal uvea may offer new insights into ocular development and the molecular drivers of UM dissemination, motility, and plasticity.

## Results

### Clustering of whole fetal uveal single-cell RNA-seq dataset reveals the presence of 14 major cell types

**Figure 1:**
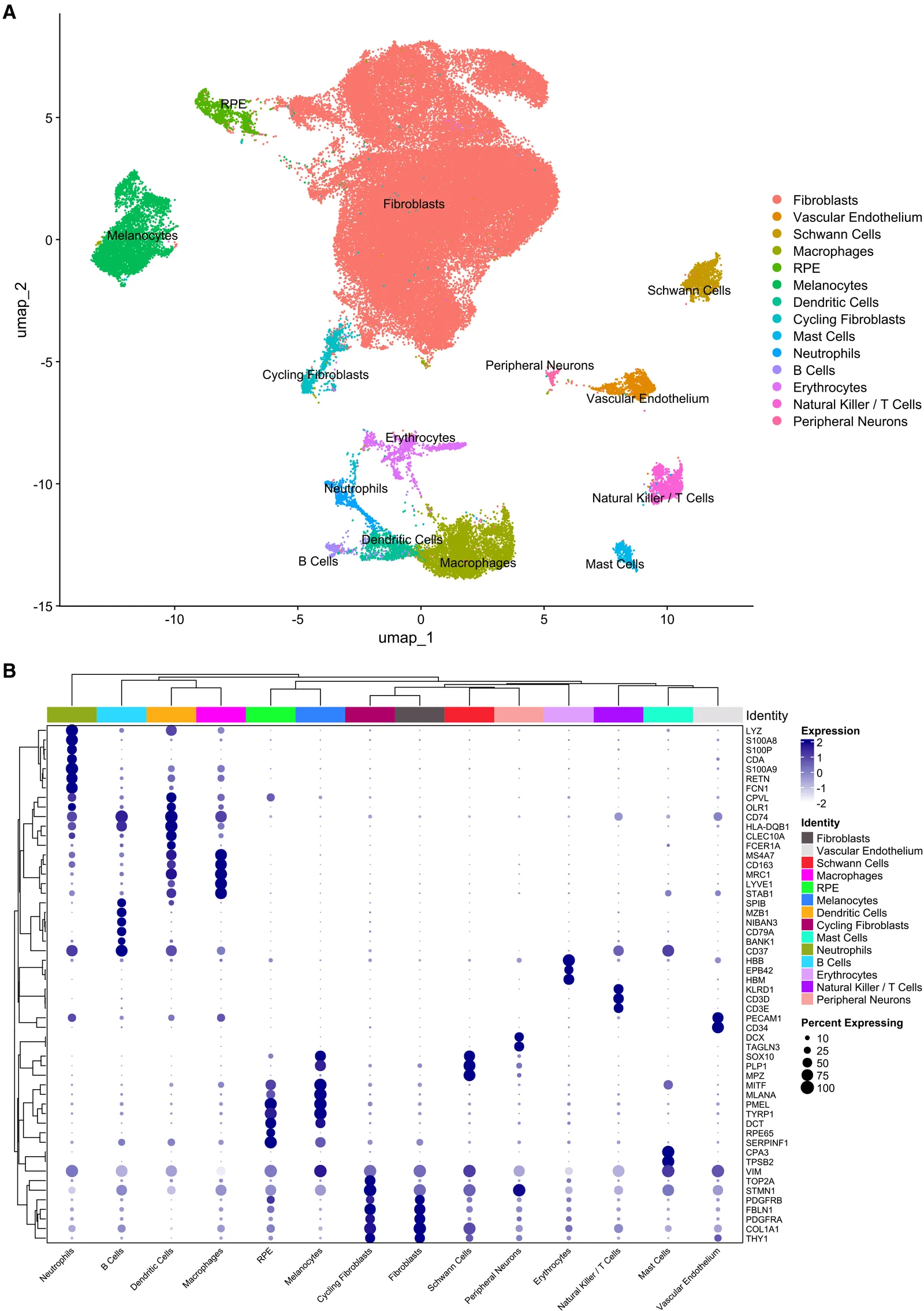
Cell-type clustering and annotation of whole fetal uveal tract atlas (A) UMAP visualization of whole fetal uveal dataset, displaying 81,603 cells grouped by annotated cell type. 14 distinct cell types were identified following clustering and marker-based annotation. (B) Clustered dot plot highlighting expression of key marker genes across annotated cell types to confirm cell-type identity. Dot size indicates the proportion of cells in a cluster expressing a given gene; opacity indicates mean relative expression intensity. Dendrograms on the *x* and *y* axes visualize hierarchical clustering driven by similarity in gene expression patterns across cell types and functional relationships between genes, respectively.

To investigate cellular heterogeneity within the fetal uveal tract, we analyzed scRNA-seq data of the whole uveal tract from 8 fetal eyes (4 × 12 PCW, 4 × 20 PCW). Each fetal uveal tract was bisected (anterior/posterior) prior to sequencing, resulting in a total of 16 datasets. Following quality control filtering, a total of 81,603 cells were identified, including 5,142 melanocytes, and retained for downstream analyses. These cells were integrated and clustered as described in the STAR Methods. During the Harmony batch correction, convergence was observed after four iterations, suggesting that batch effects were minimal in this dataset. From 17 clusters generated at Leiden resolution 0.1, subsequent review of differential expression of canonical marker genes across the clusters identified 14 major cell types, which were annotated. The uniform manifold approximation and projection (UMAP) visualization (Figure 1A) illustrates the results of clustering and annotation. To validate assigned cluster identities, a dot plot of selected marker genes (Figure 1B) was generated, highlighting cell-type-specific expression profiles. Hierarchical clustering displayed in the dot plot provides additional insight into transcriptional similarities across cell types and provides functional groupings of selected genes.

### Single-cell analysis reveals four melanocyte subpopulations within the fetal uveal tract

To investigate melanocyte heterogeneity in the developing uveal tract and explore the nature of this heterogeneity, melanocytes were isolated from the whole uveal tract Seurat object to be analyzed independently as described in STAR Methods. Within the whole dataset, 5,142 melanocytes were identified, the majority derived from 20 PCW posterior uveal tissue.

**Figure 2:**
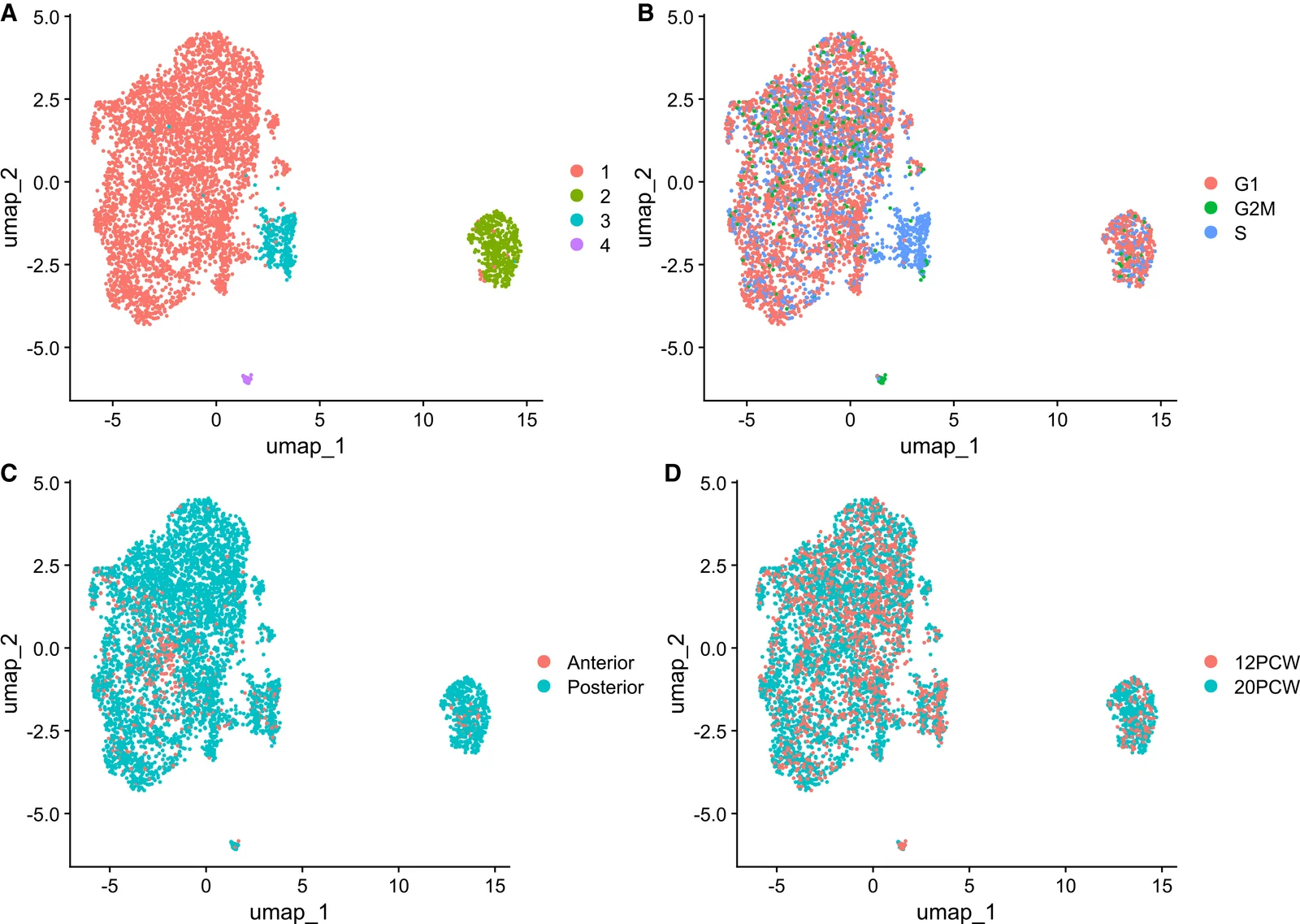
UMAP visualization of fetal melanocytes (A) Cells colored by Leiden clustering identity (resolution 0.1), reveals four transcriptionally distinct populations. (B) Cells colored by cell-cycle phase, highlights that clusters 3 and 4 are dominated by S and G2M phases, respectively, evidence that these are cycling melanocyte clusters. Clusters 1 and 2 conversely do not show any clear bias toward a particular cell-cycle phase. (C) Cells colored by uveal position (anterior vs. posterior), showing prominence of posterior section melanocytes in the total count. Though gross cell count is low anterior uveal cells still contribute to each cluster. (D) Cells colored by age (12 PCW vs. 20 PCW). Visually indicates that age is not a major driver of clustering in uveal melanocytes.

Leiden clustering at resolution 0.1 yielded 4 distinct melanocyte clusters. To evaluate the robustness of clustering, top marker gene lists were reviewed and UMAP overlays were examined by cluster identity, cell cycle phase, anatomical location, and age to assess integration of data and the influence of known variables on clustering structure (Figure 2). Across tested resolutions (0.1–0.8), clusters 2, 3, and 4 remained stable. Cluster 1 fragmented at higher resolutions, though differential expression analysis revealed minimal transcriptomic differences between these subdivisions, indicating artificial fragmentation (Figure S1). Melanocyte clusters 3 and 4 were almost exclusively occupied by cells in the S or G2M phase of the cell cycle, respectively, indicating that their cell-cycle phase was the primary driver of clustering. Differential expression analysis revealed that in these clusters, only genes associated with proliferation/cell-cycle transition are upregulated. Thus, clusters 3 and 4 will be referred to as “cycling melanocytes” collectively. Clusters 1 and 2 did not show any obvious bias when plotted for cell cycle phase, indicating that this is not a distinguishing factor in their clustering. Cell counts stratified by developmental time point, anatomical location, and cluster assignment are provided in Table 1. All four melanocyte clusters were present across each metadata condition.

**Table 1:** Melanocyte cluster distribution across developmental time point and anatomical location

	Total melanocytes	Cluster 1	Cluster 2	Cluster 3	Cluster 4
12 PCW anterior	159	134	4	18	3
12 PCW posterior	1,398	1,204	84	95	15
20 PCW anterior	464	400	33	26	6
20 PCW posterior	3,121	2,633	329	144	15

Cell counts for each melanocyte cluster stratified by post-conception week (12 vs. 20 PCW) and anatomical location (anterior vs. posterior). Most melanocytes were obtained from 20 PCW samples, reflecting greater tissue yield at later developmental stages. All four clusters are represented across all metadata conditions.

UMAPs for anatomical location and age highlighted that these metadata conditions were also not major drivers of melanocyte clustering. The 4 distinct melanocyte clusters were defined as follows: (1) canonical melanocytes (85% of total melanocytes); (3 and 4) two cycling populations (5.5% and 0.75%, respectively); and (2) a transcriptionally distinct cluster (8.75% of total melanocytes). Anterior and posterior melanocytes contributed to each cluster identified, and 12 PCW and 20 PCW melanocytes were distributed across every cluster without clear dominance according to these conditions. All clusters, including cluster 2, showed balanced representation across original donor samples without batch-driven artifacts (Figure S2).

### Identification of a transcriptionally distinct melanocyte subpopulation in the fetal uveal tract

Cluster 1 represented most melanocytes in the fetal uveal tract and exhibited a transcriptional profile consistent with canonical melanocyte identity. Cells in cluster 1 show high expression of core melanocyte markers *MLANA*, *TYRP1*, *PMEL*, *PAX3*, *MITF*, and *DCT*. Differential gene expression analysis did not identify any genes specifically upregulated in cluster 1, indicating that this population lacks distinct transcriptomic features beyond those shared across other melanocyte clusters.

**Figure 3:**
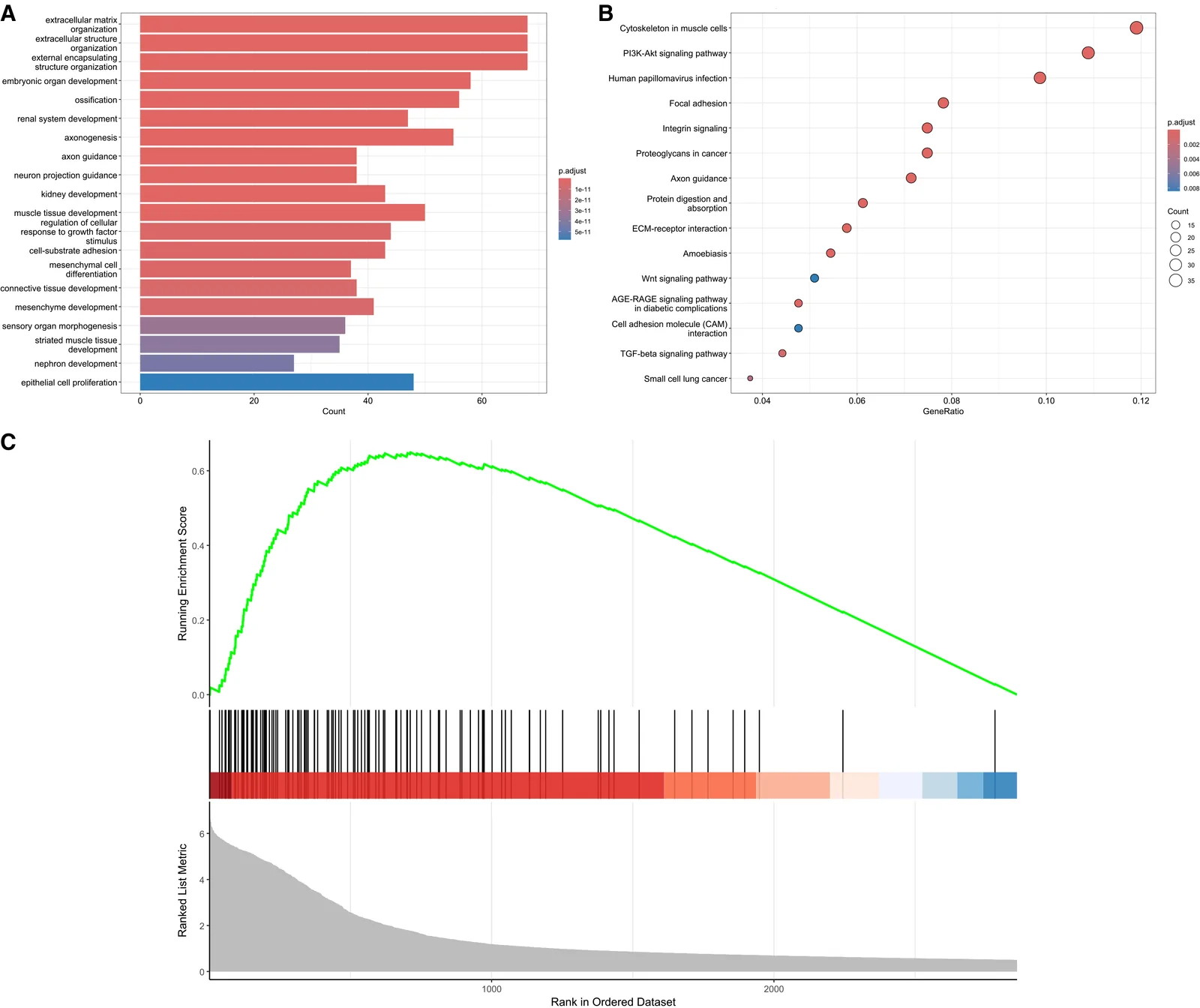
Functional enrichment analyses of cluster 2 melanocytes (A) Gene Ontology (GO) enrichment for biological processes using genes upregulated in cluster 2 (min.pct = 0.1, log_2_FC > 2) revealed significant overrepresentation of pathways related to extracellular matrix organization, morphogenesis, neural and mesenchymal development, and epithelial-to-mesenchymal transition. (B) KEGG pathway analysis of the same gene list further describes cluster 2’s phenotype, highlighting enrichment of cytoskeletal remodeling, focal adhesion, PI3k-Akt, and Wnt signaling pathways. (C) Gene set enrichment analysis (GSEA) performed on all variable genes ranked by log_2_FC revealed strong enrichment for epithelial-to-mesenchymal transition in cluster 2 as the top-scoring enriched pathway.

Cluster 2 melanocytes comprise 450 cells, accounting for 8.75% of the total melanocyte population. Although these cells retained expression of canonical melanocyte markers (Table 2), they exhibited a subtle but consistent reduction in both the percentage of cells expressing core markers and in mean expression levels, suggesting a distinct phenotype. Despite these differences, 99.8% of cluster 2 cells were triple-positive for *PMEL*, *MLANA*, and *TYRP1* (100% triple-positivity observed in clusters 1, 3, and 4), confirming robust melanocyte identity. Differential expression analysis (log_2_FC > 2, min.pct = 0.1) revealed 634 genes upregulated in cluster 2. Gene Ontology (GO) (biological process) analysis of these genes (Figure 3A) demonstrated enrichment of genes associated with extracellular matrix (ECM) remodeling, tissue morphogenesis, nervous system development, mesenchymal and neural crest development, epithelial-to-mesenchymal transition (EMT), and stemness. Kyoto Encyclopedia of Genes and Genomes (KEGG) analysis using cluster 2 provided further characterization (Figure 3B), highlighting enrichment of pathways such as cytoskeletal architecture, focal adhesion, axon guidance, PI3K-Akt signaling pathway, and Wnt signaling. Gene set enrichment analysis (GSEA) was performed (log_2_FC > 0.5, *p* > 0.05), revealing strong enrichment for EMT in cluster 2 (Figure 3C).

**Table 2:** Differential expression of canonical melanocyte markers between clusters 1 and 2

Gene name	Average log_2_FC	% of cells expressing (cluster 1)	% of cells expressing (cluster 2)	*p*_value adjusted
*MLANA*	0.61	100	100	1.48e^−109^
*TYRP1*	0.79	100	99.8	8.75e^−97^
*PMEL*	0.54	100	100	1.89e^−38^
*PAX3*	0.86	91.3	84.9	9.22e^−36^
*MITF*	0.66	99.5	98.7	4.71e^−30^
*DCT*	0.63	74.2	67.8	1.00e^−00^

**Figure 4:**
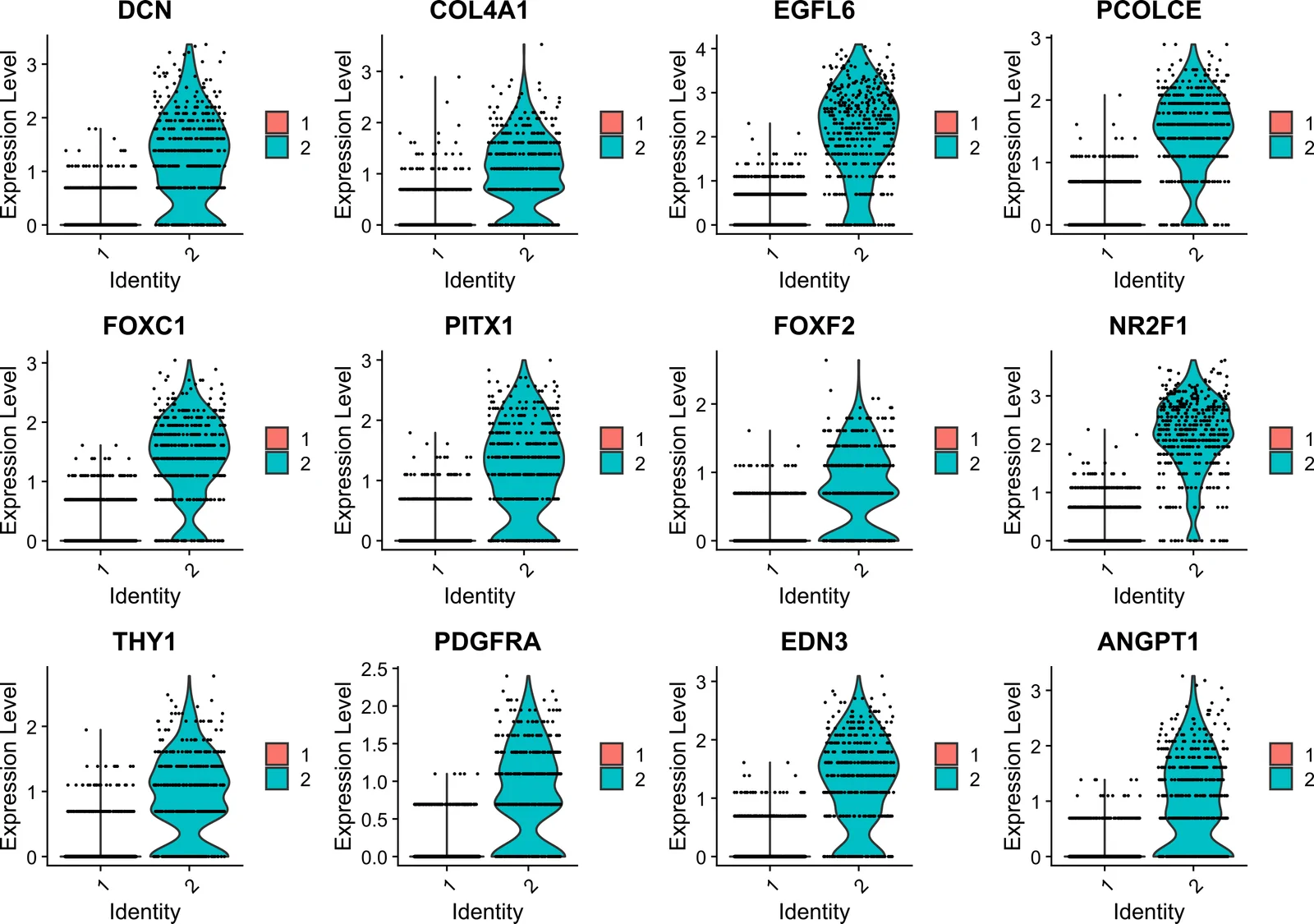
Selected high-delta genes show specific expression in melanocyte cluster 2 Violin plots showing expression of twelve top delta-ranked genes (*DCN*, *COL4A1*, *COL4A2*, *PCOLCE*, *FOXC1*, *PITX1*, *FOXF2*, *NR2F1*, *THY1*, *PDGFRA*, *EDN3*, and *ANGPT1*) across melanocyte clusters (identity 1: non-cluster 2 melanocytes; identity 2: cluster 2). Each gene displays markedly enriched or near-exclusive expression in cluster 2 relative to all other clusters, consistent with a distinct transcriptional program defining this melanocyte state.

To further define the specific signature of cluster 2, genes were ranked by Δ-expression (difference between the percentage of cluster 2 cells expressing a gene minus the percentage in all other melanocytes) (log_2_FC > 2, pct2 > 0.5, pct1 < 0.2). This approach revealed a substantial set of 114 genes with near-exclusive expression in cluster 2 (Table S1). Included amongst the genes with the highest Δ-expression scores are ECM-associated genes *DCN*, *COL4A1*, *COL4A2*, and *PCOLCE*. Developmental transcription factors such as *FOXC1*, *PITX1*, *PITX2*, *FOXF2*, and *NR2F1* also display high Δ-expression scores specific to cluster 2. Several signaling molecules, including *THY1*, *PDGFRA*, *EDN3*, and *ANGPT1*, were almost exclusively expressed among cluster 2 melanocytes. Violin plots of representative genes (Figure 4) further illustrate the distinctive transcriptomic phenotype of this population.

### Cluster 2 melanocytes are enriched in posterior, later-stage uveal tissue

**Figure 5:**
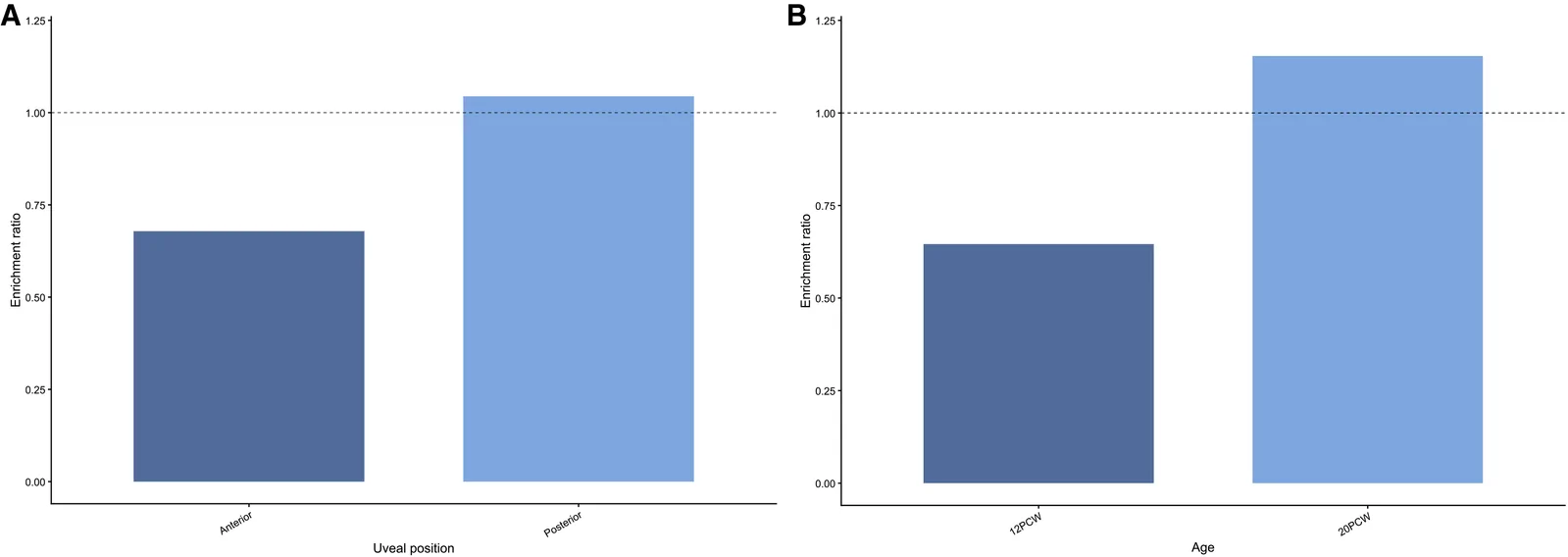
Enrichment of cluster 2 melanocytes by anatomical location and developmental age (A) Posterior uveal tract melanocytes were modestly enriched, while anterior melanocytes were underrepresented (*p* = 0.008). (B) Cells from 20 PCW tissue were enriched, while 12 PCW melanocytes were underrepresented (*p* < 0.0001). Enrichment ratios (*y* axis) were calculated as the proportion of cells from each metadata category within cluster 2 divided by their proportion in the whole melanocyte dataset. A ratio of 1.00 (dotted line) indicates proportional representation.

To investigate whether the phenotype observed in cluster 2 melanocytes reflects a developmentally immature state or an anatomically restricted population, cluster 2 enrichment ratios were quantified across developmental age and anatomical location (Figure 5). Enrichment ratios were defined as the proportion of cells from each metadata condition within cluster 2 divided by their overall proportion in the whole melanocyte dataset. This analysis showed that cluster 2 melanocytes were enriched in the posterior uveal tract (enrichment ratio = 1.04) and in 20 PCW tissue (1.15), revealing a bias to more posterior, relatively mature tissue. In contrast, cells from the anterior uveal tract (0.68) and 12 PCW tissue (0.64) were underrepresented, though still present. Both distributions displayed a significant difference from the null expectation of proportional representation (Fisher’s exact test, anatomical location, *p* = 0.008; developmental age, *p* < 0.0001), indicating a bias in cluster 2 phenotype toward posterior and 20 PCW uveal tissue.

### Intercellular communication analysis reveals a distinct signaling profile in cluster 2 melanocytes

**Figure 6:**
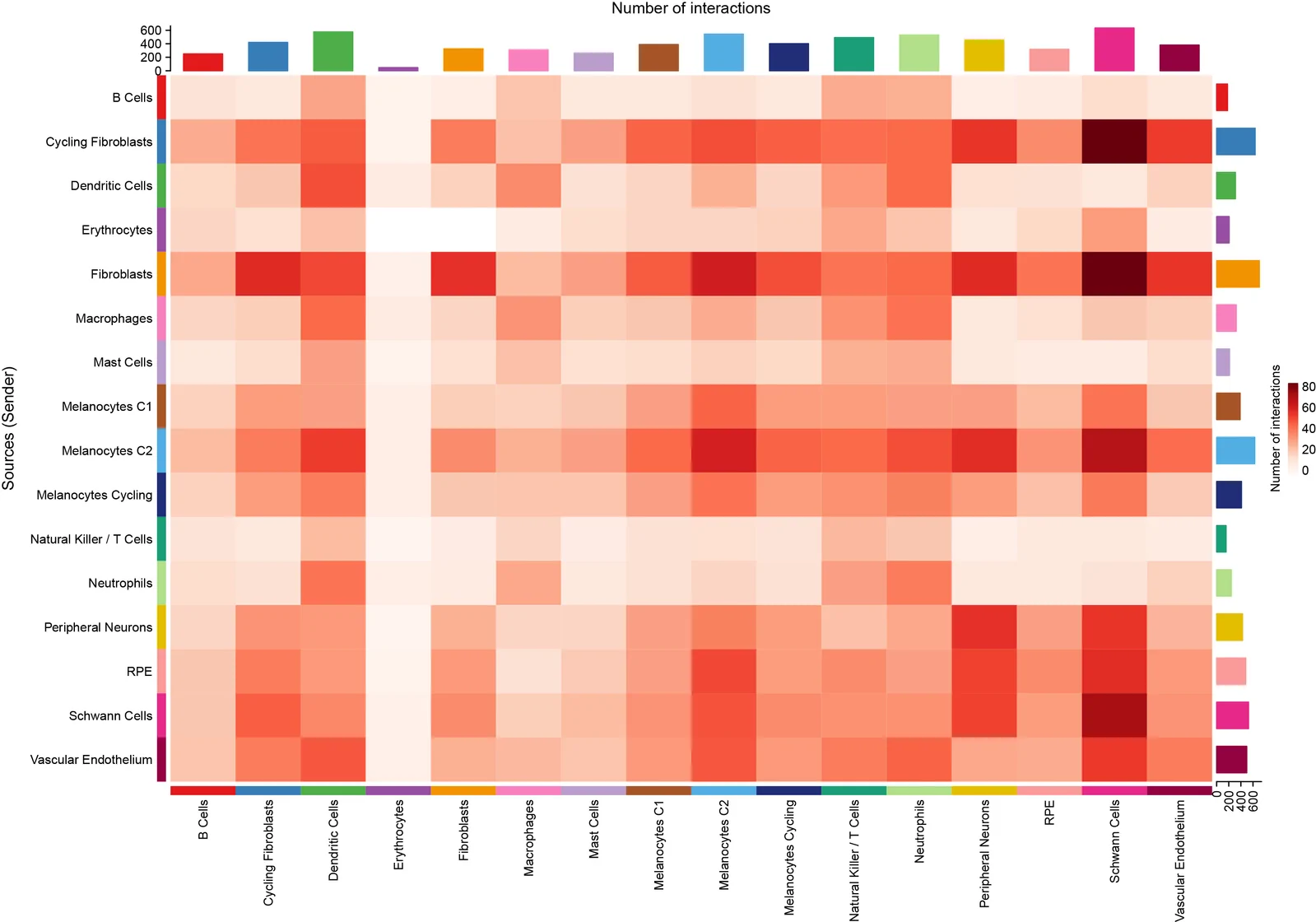
Heatmap summarizing intercellular communication across all identified cell clusters in the fetal uveal tract For interacting pairs, cells are labeled as senders or receivers. Sender cells are plotted on the *y* axis with receiver cells on the *x* axis. Squares at intersections on the heatmap reflect sum communication probability between the two cell types, with a darker color showing stronger interaction strength. Bar plots on top (above columns) and on right (end of rows) denote the total number of sender and receiver interactions identified for each cell type, respectively. This heatmap summarizes the probability of signaling within the whole dataset.

A global summary of signaling interactions across all identified cell types in the fetal uveal tract is shown in Figure 6. Cluster 2 melanocytes exhibited substantially higher interaction strength compared with other melanocyte populations, both as signaling sources and receivers. Notably, cluster 2 melanocytes showed prominent communication with dendritic cells, Schwann cells, peripheral neurons, and vascular endothelium, as well as strong autocrine signaling within the cluster itself.

**Figure 7:**
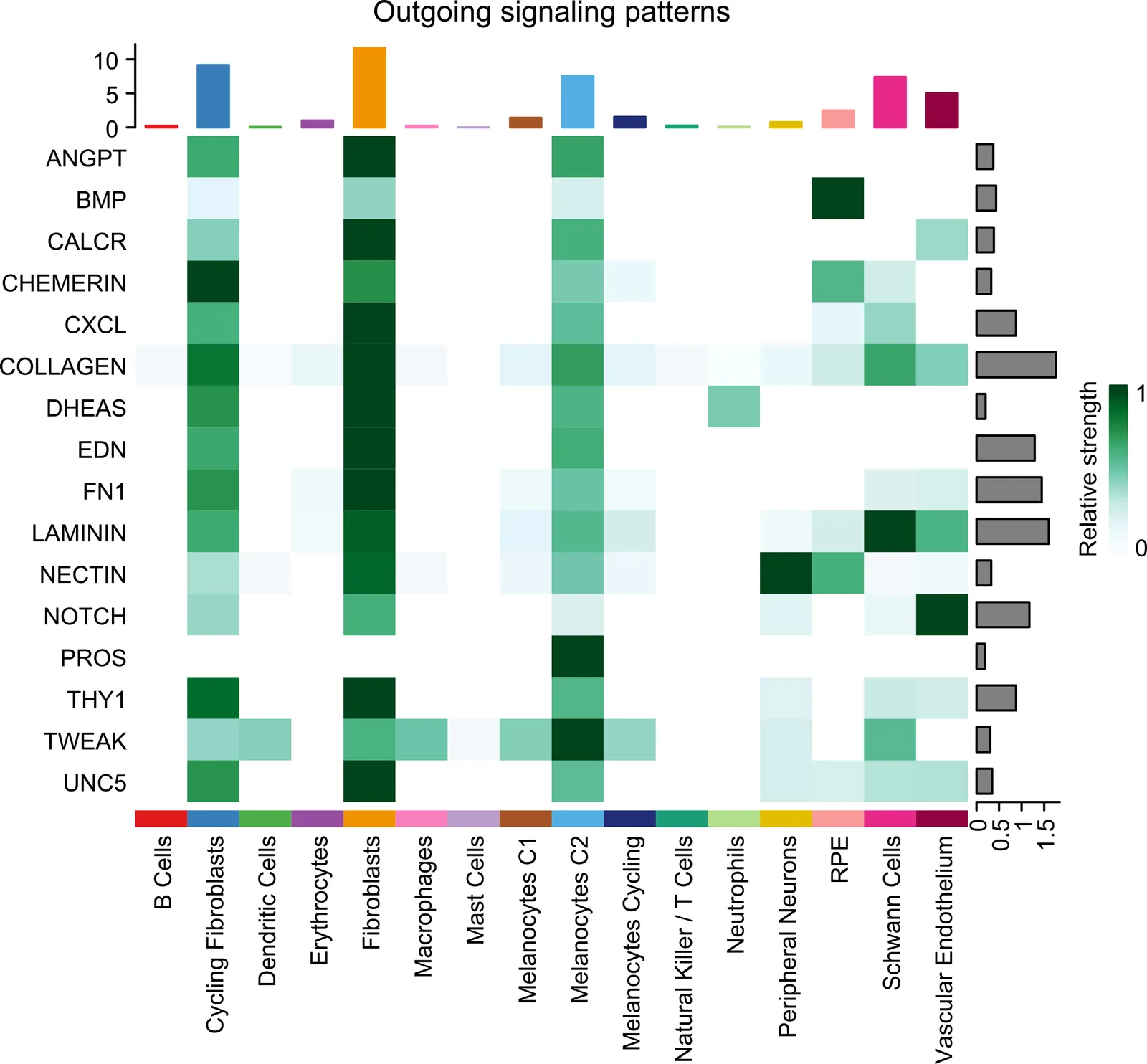
Outgoing signaling of pathways enriched in cluster 2 melanocytes Outgoing signaling role heatmap showing all CellChat pathways with significant signaling probability in cluster 2 melanocytes but not in other melanocyte populations (*ANGPT*, BMP, CALCR, *CHEMERIN*, *CXCL*, COLLAGEN, DHEAS, *EDN*, *FN1*, LAMININ, *NOTCH*, PROS, *THY1*, *TWEAK*, and *UNC5*). Row bar plots indicate cumulative outgoing signaling strength for each pathway; column bar plots indicate cumulative signaling strength for each cell type across pathways listed.

Given the expression of stromal-associated genes (*DCN*, *COL4A1*, and *THY1*) and similarity to fibroblasts in the inferred communication profile of cluster 2 (Figure 7), we performed further quality control analyses to confirm exclusion of doublets, mislabeled fibroblasts, and ambient RNA contamination. Quality control metrics, including doublet scores, UMI (unique molecular identifier)/gene counts, ribosomal and mitochondrial percentages (Table S2), confirmed the absence of technical artifacts. To directly assess whether cluster 2 cells co-express canonical melanocyte markers alongside the mesenchymal genes that define this subpopulation, paired marker co-expression was examined across all melanocyte clusters.

### Co-expression of melanocyte and stromal markers confirms cluster 2 identity

**Figure 8:**
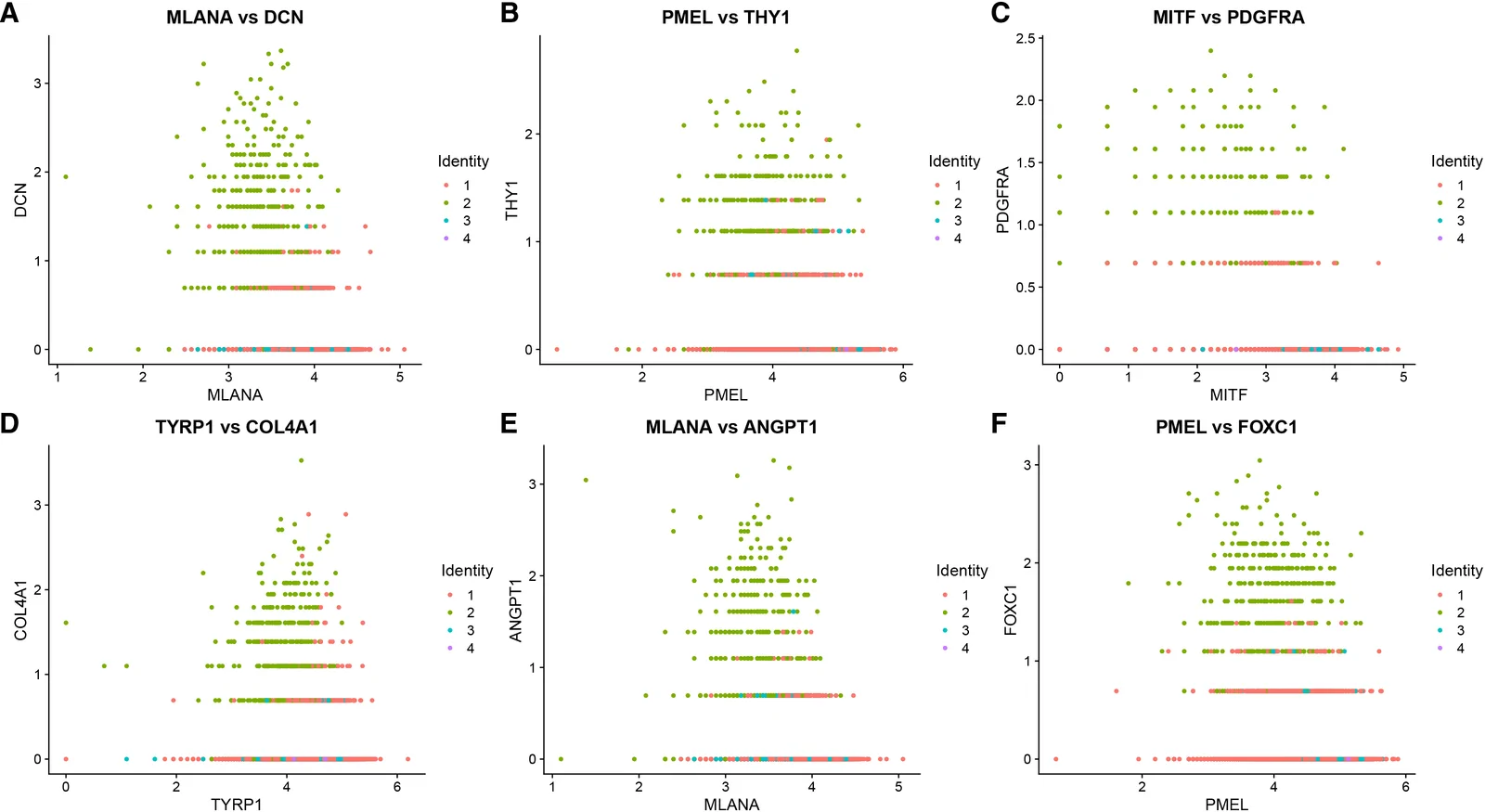
Co-expression analysis excludes fibroblast contamination in cluster 2 Scatter plots showing expression of canonical melanocyte markers (*x* axis) versus stromal-associated genes (*y* axis) across melanocyte clusters. Each dot represents an individual cell; colors indicate cluster identity (cluster 1, orange; cluster 2, green; cluster 3, blue; cluster 4, purple). (A) *MLANA* vs. *DCN*, (B) *PMEL* vs. *THY1*, (C) *MITF* vs. *PDGFRA*, (D) *TYRP1* vs. *COL4A1*, (E) *MLANA* vs. *ANGPT1*, (F) *PMEL* vs. *FOXC1*. Cluster 2 cells maintain high expression of melanocyte markers while co-expressing stromal genes, whereas other melanocyte clusters express primarily melanocyte markers with minimal stromal gene expression. Genuine fibroblast contamination would appear as a distinct cell population with high stromal marker expression and absent melanocyte marker expression; no such population is observed.

Scatterplot analysis of paired melanocytic and stromal markers across all four melanocyte clusters revealed that cluster 2 cells maintain high expression of canonical melanocyte markers (*MLANA*, *PMEL*, *MITF*, and *TYRP1*) while simultaneously expressing stromal-associated genes (*DCN*, *THY1*, *PDGFRA*, *COL4A1*, *ANGPT1*, and *FOXC1*; Figure 8; see also Figure S3). Other melanocyte clusters expressed melanocyte markers with minimal stromal gene expression. No discrete population with high stromal and low/absent melanocytic marker expression was observed; this would be expected if fibroblast contamination was present. These plots further highlight cluster 2 as a transcriptionally distinct melanocyte state.

### Spatial validation of cluster 2 melanocytes in fetal choroidal tissue

Although transcriptomic co-expression analysis confirms the identity of cluster 2 cells in the dataset, spatial validation still presents a challenge. Fetal uveal melanocytes at 12 and 20 PCW are amelanotic and several cluster 2 enriched markers, such as *FOXC1* and *NR2F1* are also expressed by surrounding stromal fibroblasts. Co-expression of a canonical melanocyte marker alongside a cluster 2 marker is required to validate this population in tissue. To address this, multiplex immunofluorescence was performed on FFPE sections from fetal eyes at 12 and 20 PCW using a 6-plex Opal panel, targeting canonical melanocyte (*MITF*), cluster 2-specific markers (*FOXC1*, *NR2F1*, and *EDNRA*), Schwann cell (*MPZ*), and endothelial (*CD31*) markers.

**Figure 9:**
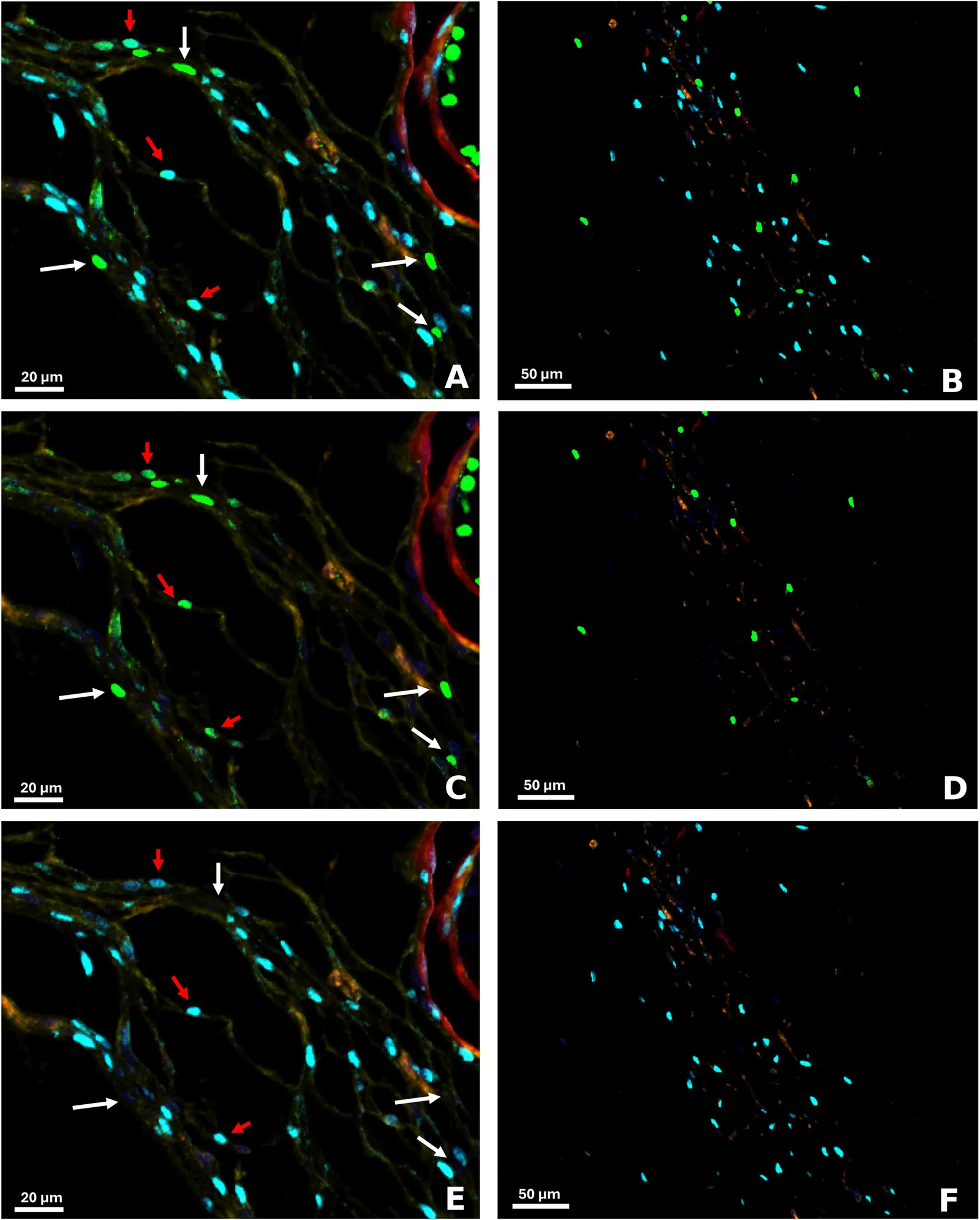
Multiplex immunofluorescence identifies MITF^+^/FOXC1^+^ co-expressing cells in fetal uveal tissue (A and B) Merged channels: MITF (green), FOXC1 (cyan), CD31 (red), MPZ (orange), EDNRA (yellow), and DAPI (blue). NR2F1 channel omitted for visual clarity. Red arrows indicate cells co-expressing MITF and FOXC1 (cluster 2 melanocytes); white arrows indicate MITF^+^/FOXC1^−^ canonical melanocytes; unlabeled cyan nuclei represent MITF^−^/FOXC1^+^ fibroblasts. (C and D) FOXC1 channel disabled. (E and F) MITF channel disabled. The absence of co-expressing cells in the right column illustrates that cluster 2 melanocytes represent a minority population. Representative multiplex immunofluorescence from a 20 PCW fetal eye section. Two choroidal regions from the same section are shown. Left column (A, C, and E): region containing cluster 2 melanocytes. Right column (B, D, and F): region lacking cluster 2 melanocytes. Scale bars: A, C, E, 20 µm; B, D, F, 50 µm.

Examination of choroidal sections revealed three distinct nuclear staining patterns, mirroring expression profiles predicted by transcriptomic analysis. *MITF*^+^/*FOXC1*^*−*^ cells representing canonical melanocytes, *MITF*^*−*^/*FOXC1*^+^ cells representing fibroblasts, and a minority population of *MITF*^+^/*FOXC1*^+^ co-expressing cells corresponding to the cluster 2 phenotype predicted from transcriptomic analysis (Figure 9). Simultaneous expression of all three populations in the same section provides evidence of cluster 2 melanocyte identity, rather than being an artifact of single-cell processing contamination. *NR2F1* immunoreactivity showed a degree nuclear co-localization with *MITF*^+^ cells; however, the signal was weak in contrast to *FOXC1*, potentially reflecting post-transcriptional regulation or differential antibody sensitivity following multiplex application. *EDNRA* staining was broadly distributed across multiple cells and could not be readily attributed to any specific subpopulation, precluding its use for discrimination here. *MPZ*^+^ Schwann cells and *CD31*^+^ endothelial cells were identified within the choroidal stroma, providing spatial context for melanocyte localization within the uveal stroma.

### Melanocyte cluster 2 program associates with molecular risk clusters

**Figure 10:**
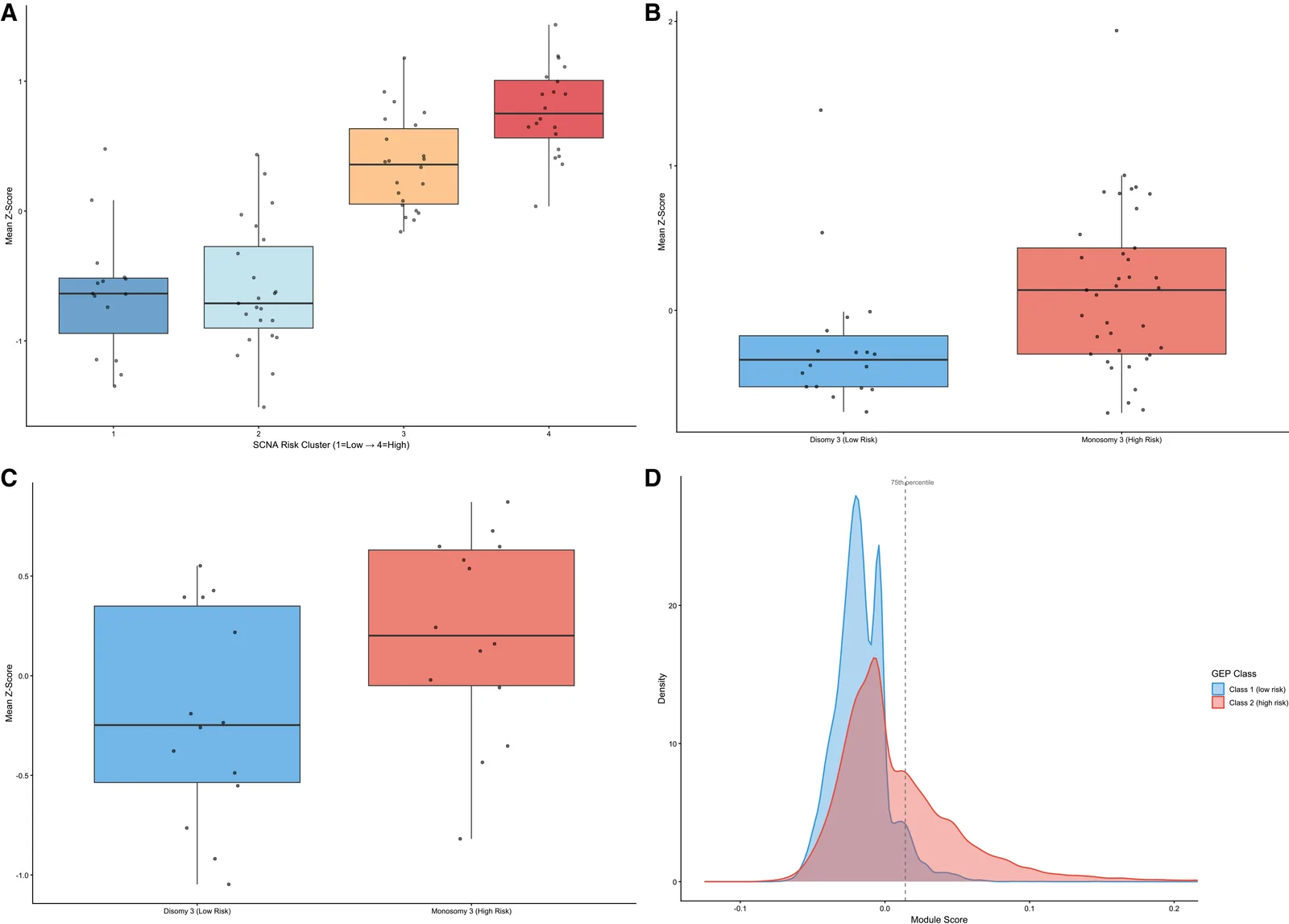
Refined cluster 2 gene signature associates with molecular risk across independent uveal melanoma cohorts (A) Boxplots showing mean *Z* scores of the refined 20-gene cluster 2 signature across SCNA risk clusters 1–4 in TCGA-UVM (*n* = 80; Spearman *ρ* = 0.782, *p* < 0.0001). (B) Validation in GSE22138 (*n* = 55, Affymetrix U133 Plus 2.0 array) stratified by chromosome 3 status (*ρ* = 0.356, *p* = 0.008). (C) Validation in GSE84976 (*n* = 28, Illumina HumanHT-12 array) stratified by chromosome 3 status (*ρ* = 0.389, *p* = 0.041). Monosomy 3 denotes high metastatic risk. (D) Density plot of refined 20-gene module scores in primary UM tumor cells from Durante et al. scRNA-seq dataset (*n* = 31,340 cells), stratified by GEP class. Dashed line indicates the 75th percentile threshold; 32.5% of class 2 cells vs. 5.5% of class 1 cells exceed this threshold (Wilcoxon *r* = 0.337). Individual boxes show median and interquartile range, dots represent individual samples (A–C).

To identify the most robustly associated genes within this program for cross-platform validation, we ranked all 171 mapped genes by their individual Spearman correlation with SCNA (Somatic copy number alteration) risk class in TCGA-UVM. The 20 most strongly associated genes comprised a coherent neural crest EMT transcriptional program (Table S4), including the transcription factors *FOXC1*, *TWIST1*, and *PRRX1*; TGFβ/BMP signaling mediators (*TGFBI*, *HTRA1*, and *FST*); Wnt pathway modulators (*SULF2*, *LPAR1*); and the top-ranked gene (*ρ* = 0.744, *p* < 0.001), *SHISA2*, a Wnt/FGF antagonist with a role in neural crest specification. This refined 20-gene signature was then tested in two independent UM cohorts profiled of distinct microarray platforms (Table S5).^25^^,^^26^ Consistent positive associations with high-risk molecular features were observed across all three datasets: TCGA-UVM (*ρ* = 0.782, *p* < 0.0001, *n* = 80), GSE22138 (*ρ* = 0.356, *p* = 0.008, *n* = 55, Affymetrix U133 Plus 2.0), and GSE84976 (*ρ* = 0.389, *p* = 0.041, *n* = 28, Illumina HumanHT-12), with higher signature scores in monosomy 3 tumors compared with disomy 3 tumors in both microarray cohorts (Figures 10A–10C). This association was maintained when ECM/collagen genes were excluded from the signature. This association was maintained when ECM/collagen genes were excluded from the signature, with the top 10 non-stromal genes alone retaining significant associations in both microarray cohorts (GSE22138 *p* = 0.050, GSE84976 *p* = 0.016), demonstrating that the observed correlation is not is not attributable to stromal contamination of bulk tumor profiles.

To further contextualize these findings at single-cell resolution, we applied the refined 20-gene signature to the Durante et al.^23^ scRNA-seq dataset. Following quality control and Seurat object processing, metastatic cells and non-melanoma cells were excluded, yielding a total of 31,340 cells from class 1 (low-risk GEP class 1) and class 2 (high-risk GEP class 2) primary tumors. Each cell was scored for the refined 20-gene signature using AddModuleScore(). Among cells in the top quartile of signature expression, class 2 cells were 6-fold more prevalent than class 1 cells (35.5% vs. 5.5%; Figure 10D). Across all primary tumor cells, class 2 cells had higher signature scores with moderate effect size (Wilcoxon *r* = 0.337, *p* < 2 × 10^−16^).

## Discussion

A long-standing paradigm has been that uveal melanocytes are homogeneous. In this study, we present a single-cell transcriptomic atlas of the human fetal uveal tract, revealing heterogeneity within developing melanocytes. Through unbiased clustering and comprehensive functional characterization, we identified four distinct melanocyte populations, including a transcriptionally distinct population (cluster 2), characterized by a mesenchymal-like gene expression program. This population exhibits hallmarks of developmental plasticity, including enrichment for ECM remodeling, cell migration, and morphogenic signaling pathways. Critically, we demonstrate that the cluster 2 gene signature correlates significantly with high-risk molecular subtypes in UM, possibly suggesting that developmental programs in these active fetal melanocytes may be recapitulated in aggressive disease states. These findings provide new molecular insights into uveal melanocyte biology and their roles in choroidal vascularization and tissue maturation and are suggestive of specific developmental cell states in UM pathogenesis.

Our analysis identified two major non-cycling melanocyte populations with distinct transcriptomic profiles. Cluster 1 exhibited canonical melanocyte identity with robust expression of *MLANA*, *TYRP1*, *PMEL*, *PAX6*, *MITF*, and *DCT*. Cluster 2 melanocytes displayed a more complex phenotype; although they retained expression of core melanocyte markers, they upregulated 634 genes associated with mesenchymal differentiation, ECM organization, and developmental morphogenesis. Importantly, a similar percentage of cells expressing canonical markers across both clusters (Table 2) argues against cluster 2 simply representing an undifferentiated or immature state. Instead, the enrichment of cluster 2 in posterior uveal tissue and at a later developmental time point (20 PCW) suggests this population expands during ocular maturation, potentially reflecting functional specialization rather than developmental delay.

Among the features more distinctive in cluster 2 were genes involved in ECM remodeling (*DCN*, *COL4A1*, *COL4A2*, and *PCOLCE*), developmental transcription factors (*FOXC1*, *PITX1*, *PITX2*, *FOXF2*, and *NR2F1*), and signaling molecules (*THY1*, *PDGFRA*, *EDN3*, and *ANGPT1*) showing near-exclusive expression in this population (Table S1). GO highlighted mesenchymal differentiation, connective tissue development, sensory organ morphogenesis, and axon guidance—themes involving large gene complements (40–60 genes each) passing stringent log_2_ fold change thresholds, supporting their biological relevance. KEGG analysis identified PI3K–Akt signaling, ECM-receptor interaction, and TGF-β pathways—canonical regulators of melanocyte survival, motility, and microenvironmental interactions.^22^^,^^27^ GSEA further corroborated these findings, demonstrating strong enrichment for EMT. Together, these findings suggest that cluster 2 melanocytes retain or acquire a transcriptional state geared toward tissue remodeling and morphogenesis, reflecting developmental plasticity that extends beyond pigment production.

Computational cell-cell communication analysis provided additional functional context for cluster 2 melanocytes. These cells ranked among the most prominent signaling sources in the fetal uveal tract, second only to the two fibroblast populations in outgoing signaling strength. Notably, cluster 2 melanocytes communicated along pathways that were absent or minimal in the other melanocyte populations, functioning as near-exclusive melanocytic sources of *THY1*, endothelin (EDN), CXCL chemokine, and angiopoietin (ANGPT) pathway signaling, amongst several other pathways. Despite robust expression of canonical melanocyte markers (Table 2), this signaling repertoire closely resembled that of fibroblasts, suggesting cluster 2 melanocytes may have a role in stromal remodeling and tissue architecture during uveal development.

Expression of collagens, laminin, *THY1*, and EDN pathway signaling implicates these cells in functions in the traditional domain of fibroblasts. EDN signaling, mediated primarily through *EDN3*, has established roles in neural crest cell migration and melanocyte development,^22^^,^^28^^,^^29^ and its expression by cluster 2 melanocytes may facilitate continued tissue remodeling during uveal maturation. Similarly, *THY1* (CD90) is a marker of mesenchymal stem cells and has been implicated in cell adhesion, migration, and fibroblast activation,^30^^,^^31^ further supporting a stromal modeling function for this population.

Cluster 2 melanocytes also exhibit an immunomodulatory signaling profile, expressing chemokines and cytokines, including *CHEMERIN*, *TWEAK*, and CXCL pathway ligands. Chemerin is a chemoattractant adipokine that recruits immune cells, particularly macrophages and dendritic cells, and has been shown to modulate inflammatory responses.^32^^,^^33^ *TWEAK* (TNF-like weak inducer of apoptosis) signals through the *FN14* receptor to regulate tissue remodeling, angiogenesis, and immune cell recruitment during development and injury repair.^34^^,^^35^ The expression of CXCL chemokines further positions cluster 2 melanocytes as coordinators of immune cell trafficking within the developing uveal stroma. Given that computational inference predicted communication between cluster 2 melanocytes and dendritic cells, neutrophils, and natural killer/T cells, this immunomodulatory signature may reflect an active role in establishing immune surveillance mechanisms within the fetal uveal tract.

Perhaps most notably, cluster 2 melanocytes express key vasculogenic factors, particularly *ANGPT1* and components of the UNC5 signaling pathway. *ANGPT1* is a critical regulator of vascular maturation and stability, promoting endothelial cell survival, and vessel integrity.^36^^,^^37^ Neural crest-derived cells have been shown to be essential sources of *ANGPT1* during vascular development of the choroid.^8^ Previous studies have suggested that choroidal melanocytes are critical for proper choroidal vascular development,^7^ though the molecular mechanisms remain poorly defined. Our identification of *ANGPT1*-expressing melanocytes provides a candidate molecular basis for this relationship. Additionally, cluster 2 melanocytes are predicted to communicate with endothelium through the *FLRT2*-*UNC5B* interaction, which is also implicated in vascular development.^38^

Collectively, these findings indicate that cluster 2 melanocytes may occupy a specialized niche within the developing uveal microenvironment, with functions extending to tissue remodeling, vascular support, and immune modulation.

A central motivation for characterizing fetal uveal melanocyte heterogeneity was our hypothesis that developmental transcriptional programs may be reactivated in aggressive UM. The full 190-gene cluster 2 signature (Figure S4) showed a significant positive correlation with SCNA-based molecular risk groups in the TCGA-UVM cohort (Spearman *ρ* = 0.407, *p* = 0.0002; Kruskal-Wallis *p* = 0.001; Figure S5), with high-risk tumors displaying markedly elevated signature activity. To identify the most robustly associated genes and enable cross-platform validation, individual genes were ranked by their correlation with SCNA risk class, yielding a refined 20-gene signature with a consistent neural crest EMT identity. The refined signature demonstrated substantially stronger association in the discovery cohort, with *ρ* = 0.782 in TCGA-UVM and validated consistently in two independent microarray cohorts (GSE22138: *ρ* = 0.356, *p* = 0.008; GSE84976: *ρ* = 0.389, *p* = 0.041). Notably, this association was maintained following exclusion of ECM/stromal genes, suggesting this signal reflects tumor-intrinsic biology rather than stromal contamination of bulk tumor profiles. These findings link the transcriptional programs of fetal cluster 2 melanocytes to aggressive UM phenotypes, suggesting that the migratory and ECM-remodeling properties of this population may be co-opted in disease potentially facilitating dissemination and microenvironmental adaptation. EMT and related plasticity programs are well established drivers of metastasis and therapy resistance across cancer types,^39^^,^^40^^,^^41^^,^^42^ and the enrichment of EMT, PI3K-Akt, TGF-β, and focal adhesion pathways in cluster 2 aligns closely with known mechanisms of UM progression and liver metastasis.^43^^,^^44^^,^^45^^,^^46^^,^^47^

The composition of the refined 20-gene signature is biologically informative. The top-ranked individual gene, *SHISA2* (*ρ* = 0.744), is a Wnt/FGF antagonist with an established role in neural crest specification, suggesting that perturbation of early developmental signaling pathways may contribute to aggressive UM behavior. The transcription factors *FOXC1*, *TWIST1*, and *PRRX1* were also among the most strongly SCNA-associated genes, *TWIST1* and *PRRX1* are established drivers of EMT and metastatic invasion across cancer types,^48^^,^^49^^,^^50^^,^^51^ both *TWIST1* and *PRRX1* have previously been directly implicated in tumor progression in UM.^47^^,^^52^ Developmental transcription factor *FOXC1* is also implicated in a series of processes across several cancers, including angiogenesis, invasion, EMT, metastasis, and poor prognosis when highly expressed.^53^^,^^54^^,^^55^^,^^56^^,^^57^ Among the broader cluster 2 marker genes not captured by the refined signature, *NR2F1* is noteworthy, showing the highest delta expression among cluster 2 markers (96% of cells in cluster 2 vs. 13% in cluster 1, average log_2_FC = 5.98), and has recently been implicated in dormancy and subsequent reactivation of disseminated UM cells in the liver.^58^ This direct link between a defining feature of fetal cluster 2 melanocytes and metastatic UM behavior supports the hypothesis that developmental programs are co-opted during tumor dissemination. Together, these findings suggest that reactivation of this developmental transcriptional program may facilitate both dissemination and subsequent metastatic colonization in UM.

Our findings connect this developmental atlas directly to the transcriptional heterogeneity characterized in UM at single-cell resolution. Pandiani et al. identified distinct transcriptional cell states in primary UM, including a poor-prognosis invasive subpopulation driven by *HES6*,^22^ while Durante et al. and Li et al. independently demonstrated that malignant UM cells exist in transcriptionally distinct states with differential prognosis and immune microenvironments.^23^^,^^24^ Direct validation of the refined 20-gene signature in the Durante et al. dataset revealed significant enrichment in class 2 tumor cells relative to class 1 (35.5% vs. 5.5% of cells scoring above the 75^th^ percentile threshold; Wilcoxon *r* = 0.337, *p* < 2 × 10^−16^), indicating that the program of fetal cluster 2 melanocytes is preferentially expressed in the aggressive UM cell state. This convergence between a fetal developmental cell state and an established tumor transcriptional program supports the hypothesis that class 2 UM identity may reflect reactivation of a developmental plasticity program.

The stromal and vascular functions of cluster 2 melanocytes likely reflect adaptations to the unique uveal microenvironment. Unlike cutaneous melanocytes, which reside in a relatively avascular epidermis focused on photoprotection, uveal melanocytes occupy a densely vascularized stromal compartment where contributions to tissue architecture, angiogenesis, and immune regulation may be equally important to pigmentation. The expression of *ANGPT1*, collagens, and immunomodulatory chemokines by cluster 2 aligns with these tissue-specific demands.

This study provides a comprehensive single-cell characterization of human fetal uveal melanocytes and reveals a transcriptionally distinct subpopulation with mesenchymal, migratory, and stromal remodeling properties. Cluster 2 melanocytes exhibit extensive cell-cell communication networks, express factors critical for vascular development, and are enriched in the posterior uveal tract during later fetal stages. Importantly, the cluster 2 gene signature correlates with high-risk molecular subtypes in UM, suggesting that developmental plasticity programs may be reactivated to drive tumor progression and metastasis. These findings enhance our understanding of uveal melanocyte biology, provide new insights into ocular development, and identify candidate pathways for therapeutic intervention in UM. More broadly, this work underscores the utility of developmental atlases in illuminating cancer cell states and highlights the importance of tissue-specific context in interpreting cellular heterogeneity.

These findings open several directions for further investigation. First, spatial transcriptomics applied to fetal and adult uveal tissue, as well as primary and metastatic UM specimens, would build on the multiplex immunofluorescence data presented here to provide higher-resolution spatial context, confirm anatomical localization of melanocyte subpopulations, and reveal their spatial relationships with vasculature and immune populations within the tumor microenvironment. Second, *in vivo* knockdown or overexpression of key cluster 2 genes associated with high-risk UM, such as *FOXC1*, *PRRX1*, or *SULF2*, in preclinical UM models could establish their causal role in metastasis and identify potential therapeutic targets. Third, direct single-cell profiling of primary UM tumors and matched metastatic lesions would clarify whether the dynamics of cluster 2-like cells during metastatic progression, extending beyond signature-level validation presented here. Finally, comparative analyses across other neural crest-derived malignancies, such as neuroblastoma or cutaneous melanoma, could identify conserved developmental plasticity mechanisms that drive aggressive disease across tumor types.

### Limitations of the study

Several limitations of our study should be acknowledged. First, our analysis is derived from two fetal time points (12 and 20 PCW) and does not include postnatal or adult uveal tissue, limiting our understanding of the full temporal dynamics of melanocyte migration and differentiation, leaving open the question whether the cluster 2 state persists into adulthood or represents a transient developmental phenotype. Earlier, later, and postnatal time points would provide a more complete developmental trajectory. Second, while multiplex immunofluorescence confirmed the existence of *MITF*^+^/*FOXC1*^+^ co-expressing cells in fetal choroidal tissue, this analysis is restricted to a two-dimensional plane in a dynamic three-dimensional structure, and no quantification of subpopulation frequency or spatial distributions was performed. Whether melanocyte subpopulations show preferential distribution along specific anatomical axes cannot be determined from the current data. Spatial transcriptomics would provide more comprehensive *in situ* characterization of melanocyte subpopulations and their microenvironmental context. Third, our analysis of UM is conducted exclusively in external datasets; we do not profile primary UM tissue directly in this study. Fourth, our cell-cell communication analysis is based on ligand-receptor inference and requires experimental validation through co-culture systems, organoid models, or *in vivo* perturbation studies. Finally, the association between the cluster 2 gene signature and high-risk UM is correlative. Functional studies, such as overexpression or knockdown of key cluster 2 genes in UM cell lines or patient-derived xenografts, would provide further insight into the causal role of these programs in tumor progression and their therapeutic potential.

## Resource availability

### Lead contact

Further information and requests for resources should be directed to and will be fulfilled by the lead contact, James Draper (j.draper@liverpool.ac.uk).

### Materials availability

This study did not generate new unique reagents.

### Data and code availability

• Single-cell RNA sequencing data reported in this paper, including raw FASTQ files and processed count matrices, have been deposited at NCBI’s Gene Expression Omnibus (GEO) and are publicly available as of the date of publication under accession number GEO: GSE325954. TCGA-UVM data were accessed through the NCI Genomic Data Commons and are publicly available. External validation datasets GSE22138 and GSE84976 are publicly available through NCBI GEO. The Durante et al. uveal melanoma single-cell dataset is publicly available through NCBI GEO under accession number GEO: GSE139829. All accession numbers are listed in the key resources table.
• This paper does not report original code.
• Any additional information required to reanalyze the data reported in this paper is available from the lead contact upon request.

## Acknowledgments

The funding for this study was provided by Wendy’s Wish (registered charity 1175368) and Kennedy Trust of Rheumatology (registered charity 260059).

## Author contributions

J.D., bioinformatic experimentation and analysis and drafting of manuscript. I.R.R., generation of single-cell data, concept development, and review of the paper. A.D.D., M.C., L.W., S.S., and C.B., study conceptualization and critical review of manuscript. H.K. and S.E.C., supervision of experimentation, drafting of manuscript, and critical review of work completed.

## Declaration of interests

The authors declare no competing interests.

## STAR★Methods

### Key resources table

**Table:** 

REAGENT or RESOURCE	SOURCE	IDENTIFIER
**Antibodies**		

Anti-MITF Clone D5 Mouse Monoclonal	DAKO (Agilent)	Cat# M3621; Dilution 1:100; RRID: AB_2142100
Anti-CD31 Clone JC70A Mouse Monoclonal	DAKO (Agilent)	Cat# M0823; Dilution 1:100; RRID: AB_2114471
Anti-MPZ Clone EPR20383 Rabbit Monoclonal	Abcam	Cat# Ab183868; Dilution 1:200; RRID: AB_2895675
Anti-FOXC1 Clone EPR20685 Rabbit Monoclonal	Abcam	Cat# Ab227977; Dilution 1:500; RRID: AB_2916124
Anti-EDNRA Clone UMB-8-27-1 Rabbit Monoclonal	Abcam	Cat# Ab178454; Dilution 1:100
Anti-NR2F1 Rabbit Polyclonal	Proteintech	Cat# 24573-1-AP; Dilution 1:200; RRID: AB_2879616

**Biological samples**		

FFPE fetal eye blocks	Human Developmental Biology Resource (HDBR)	REC: 23/NE/0135
Fresh fetal eyes	Human Developmental Biology Resource	REC: 18/LO/0822 & 18/NE/0290

**Chemicals, peptides, and recombinant proteins**		

Liberase TL	Roche	Cat# 5401020001
DNAase I	Roche	Cat# 03724778103

**Critical commercial assays**		

Chromium Next GEM Single Cel 3′ Reagent Kit v3.1	10× Genomics	Cat# PN-1000268
Opal 6-Plex Detection Kit	Akoya Biosciences	Cat# NEL821001KT

**Deposited data**		

Raw and processed single-cell RNA-seq data	This paper	GEO: GSE325954
Raw and processed single-cell RNA-seq data	Durante et al.^23^	GEO: GSE139829
Microarray expression profiling data	Laurent et al.^25^	GEO: GSE22138
Microarray expression profiling data	van Essen et al.^26^	GEO: GSE84976
TCGA-UVM bulk RNA-seq data	NCI Genomic Data Commons	Project: TCGA-UVM

**Software and algorithms**		

RStudio	Posit	posit.co; RRID:SCR_000432
Cell Ranger Pipeline (v7.0)	10× Genomics	10xgenomics.com; RRID:SCR_017344
Seurat R Package (v.5.3.0)	Sajita Lab	satijalab.org; RRID:SCR_007322
DoubletFinder R package (v.2.0.4)	McGinnis et al.^59^	Github.com
Harmony R package (v1.2.4)	Korunskky et al.^60^	Github.com
clusterProfilier R package (v.4.12.6)	Yu et al.^50^	bioconductor.org; RRID:SCR_016884
MSigDB Hallmark Gene Sets (v2024.1)	UC San Diego/Broad Institute	gsea-msigdb.org; RRID:SCR_016863
CellChat R Package (v2.2.0)	Jin et al.^61^	Github.com
Akoya inForm Software (v3.0.0)	Akoya Biosciences	akoyabio.com; RRID:SCR_019155
QuPath Software (v0.6.0)	Bankhead et al.^62^	github.io; RRID:SCR_018257

### Experimental model and study participant details

Karyotypically normal, healthy fetal eyes were obtained from the Human Developmental Biology Resource (HDBR, ethical approval REC: 18/LO/0822 & 18/NE/0290). In total, eight donors were included across two developmental timepoints: four at 12 post-conception weeks (PCW; two male and two female) and four at 20 PCW (four female). FFPE fetal eye blocks for multiplex immunofluorescence were obtained from HDBR (ethical approval REC: 23/NE/0135).

### Method details

#### Tissue dissociation and single-cell library preparation

Fetal eyes were dissected to expose the uveal tract, removing non-uveal ocular tissue. Eyes were then bisected into anterior (iris and ciliary body) and posterior (choroid) uvea, using the ora serrata as the boundary.

Tissue was mechanically dissociated with scissors, then incubated at 37^0^C on a cell shaker at 300rpm with a cocktail of 0.1 mg/mL Liberase TL and 0.5 mg/mL DNAse I in RPMI 1640 medium. Once complete tissue dissociation was achieved, the single-cell suspension was filtered through a 70 μm cell strainer. Cells were re-suspended in RPMI 1640 medium +10% heat-inactivated fetal bovine serum (FBS). Cell concentration and viability were assessed using trypan blue staining prior to library preparation. Single-cell capture, barcoding, and library preparation was performed according to the Chromium Next GEM Single Cell 3ʹ Reagent Kit v3.1 protocol. Sequencing was carried out on a NovaSeq6000 sequencer. Sequences were aligned to the GRCh38 reference genome using the Cell Ranger pipeline. The resultant feature-barcode matrices were exported for downstream bioinformatic analysis.

#### Data processing

Processing of the single-cell transcriptomic data was primarily performed in RStudio (R language 4.4.0–4.4.1) using the Seurat^63^ (v.5.3.0) pipeline. Filtered cell ranger features, barcodes and matrix files were imported to R. Additional filtering of low-quality cells was performed by excluding cells with <1000 unique molecular identifiers (UMIs), <500 genes detected, and >12.5% mitochondrial DNA. Doublets were identified and excluded using the DoubletFinder(v.2.0.4) package,^59^ DoubletFinder was run with PCs = 1:20, pN = 0.25, with optimized pK values calculated per sample, and nExp calculated based on Poisson distribution expectations (∼0.8% doublet rate per 1,000 cells). Data normalisation was performed using SCTransform() (top 3000 variable features). Following this, Principal Component Analysis (PCA) was performed using RunPCA() and Uniform Manifold Approximation and Projection (UMAP) was constructed using the first 30 principal components. Cell cycle scores were calculated using CellCycleScoring(). Batch correction was performed using the Harmony package(v.1.2.4)^60^ on SCT-corrected principal components, correcting for original sample, converging in 4 iterations. Shared-nearest-neighbour graphs were constructed with FindNeighbours(). Clustering was performed using the Leiden algorithm with function FindClusters() across 9 resolutions (0.1–0.8). Each resolution was projected onto the UMAP embedding to assess cluster stability and relevance. Resolution 0.1 was selected, highlighting broad cell clusters. Within this, three clusters were subclustered using FindSubCluster(), yielding a total of 18 distinct clusters in the whole fetal uveal dataset. Cluster annotation was performed manually by examining top marker genes identified with FindAllMarkers() and comparing them to canonical cell-type markers.

#### Identification of melanocyte subpopulations

For melanocytes, raw counts were extracted from the global Seurat object using the subset() function. PCA and UMAP were recalculated, and nearest neighbor graphs were constructed using the first 30 PCs. Clustering was performed using the FindClusters() function with the Leiden algorithm across multiple resolutions, with stable clustering obtained at resolution 0.1. Differentially expressed genes for each melanocyte cluster were identified with FindAllMarkers() with Wilcoxon rank-sum test (log_2_ fold-change threshold >2, min.pct = 0.1).

#### Functional characterisation of Cluster 2

Functional enrichment was performed using ClusterProfiler (v4.12.6)^64^ with the org.Hs.e.g.,.db annotation database. Gene Ontology (Biological Process) and KEGG pathway enrichment analyses were conducted by first converting gene symbols to Entrez IDs with bitr(). Enrichment was assessed with enrichGO() (ontology = BP, Benjamini–Hochberg-adjusted q < 0.05) and enrichKEGG() (organism = “hsa”, adjusted *p* < 0.05). For GSEA, all genes were ranked by average log_2_ fold-change, and enrichment was tested against the MSigDB Hallmark gene sets (v2024.1) using GSEA() (minGSSize = 10, maxGSSize = 500, adjusted *p* < 0.05, Benjamini–Hochberg correction).

#### Cell-cell communication analysis

Cell-cell communication analysis was performed using CellChat (v2.2.0)^61^ in R, following the standard workflow outlined in the package vignette. Following identification of distinct melanocyte clusters, these were labeled and incorporated into the annotation of the global Seurat object containing all uveal tract cells. The “RNA” assay of this whole uveal tract object was normalised using NormalizeData(). A CellChat object was created using createCellChat(). The CellChat human ligand-receptor database was applied. Overexpressed genes and interactions were identified by identifyOverExpressedGenes() and identifyOverExpressedInteractions(). Communication probabilities were calculated with computeCommunProb(), low abundance interactions were filtered with filterCommunication() (min.cells = 10). Communication probabilities were then inferred at the signaling pathway level using computeCommunProbPathway(), and aggregated cell–cell communication networks were calculated with aggregateNet(). Strength of cell-cell interactions in the whole fetal uvea was visualised by heatmap with netVisual_heatmap(). Outgoing and incoming signaling heatmaps for selected pathways were generated with netAnalysis_signalingRole_heatmap() and signaling role networks were generated with netAnalysis_signalingRole_network().

#### Multiplex immunofluorescence

Formalin-fixed paraffin-embedded (FFPE) fetal eye blocks were obtained from HDBR (ethical approval REC: 23/NE/0135). Eyes from three donors at each gestational age (12 PCW and 20 PCW) were sectioned on the sagittal plane to the orbital midline to provide a cross-section of the whole fetal uveal tract, representing both anterior (iris/ciliary body) and posterior (choroid) segments. Sections were cut to 4 μm.

Multiplex immunofluorescence was performed using the Opal 6-plex Detection Kit (Akoya). The panel comprised six markers selected for their specificity of expression in cluster 2 melanocytes following DEG analysis: *MITF* (Opal 520, green; pan-melanocyte marker), *FOXC1* (Opal 480, cyan; Cluster 2 transcription factor), *NR2F1* (Opal 780, pink; Cluster 2 transcription factor), *EDNRA* (Opal 570, yellow; Cluster 2 signaling marker), *MPZ* (Opal 620, orange; Schwann cell marker) and *CD31* (Opal 690, red; endothelial marker). DAPI was used for nuclear counterstain. Antigen retrieval (pH 9.0 for all antibodies) and antibody incubation were performed sequentially for each marker following the Opal Multiplex protocol. Slides were scanned using the PhenoImager slide scanner (Akoya) at 20× magnification. Autofluorescence removal and spectral unmixing was performed using Akoya inForm (v3.0.0) software. Images were visually inspected using QuPath (v.0.6.0)^64^.

#### Gene signature scoring against bulk RNA-seq UM risk clusters

TCGA-UVM bulk RNA-seq data (80 primary UM samples) were obtained from the TCGA data portal, including RSEM-normalized gene expression values (20,501 genes) and molecular risk cluster classifications from Robertson et al. (2017), including somatic copy number alteration (SCNA) risk classes (1–4). Differentially expressed genes for cluster 2 melanocytes were generated using Seurat’s FindAllMarkers as previously described and trimmed by the following criteria: average log_2_ fold-change ≥0.5, delta percent expression (pct.1 - pct.2) ≥ 0.25, expressed in ≥20% of Cluster 2 cells (pct.1 ≥ 0.20), and expressed in ≤10% of other clusters (pct.2 ≤ 0.10), yielding a 190-gene signature highly specific to Cluster 2 (Table S3). Gene symbols were matched against TCGA expression matrix, resulting in 171 genes (90% mapping rate). Gene signature activity was quantified using mean *Z* score methodology. For each sample, individual gene expression values were *Z* score normalized across all samples and the module score was calculated as the mean of z-scores across all signature genes. Association between module scores and molecular risk clusters was assessed using the Spearman’s rank correlation coefficient and the Kruskal-Wallis test.

To identify which genes most robustly associate with UM risk classification, each of the 171 mapped genes was individually correlated with SCNA risk classification in TCGA-UVM using Spearman’s rank correlation. Genes were ranked by correlation coefficient, and the top 20 most strongly associated genes were selected as a refined signature for independent validation.

The refined 20-gene signature was validated in two independent publicly available UM microarray datasets: GSE22138 (63 primary UM samples, Affymetrix HG-U133 Plus 2.0 array)^25^ and GSE84976 (28 primary UM samples, Illumina HumanHT-12 v.40 BeadChip),^26^ downloaded using the GEOquery package (v2.72.0). Probe-level expression values were mapped to gene symbols using platform annotation files. Gene expression values were *Z* score normalised across samples and mean signature scores calculated as described above. In GSE22138, eight samples were excluded as chromosome 3 status was not included in the annotations (*n* = 55 analyzed). Association between signature scores and chromosome 3 status (disomy vs. monosomy) was assessed using Spearman’s rank correlation and the Wilcoxon rank-sum test.

To contextualise findings at single-cell resolution, the refined 20-gene signature was applied to a UM scRNA-seq dataset GSE139829.^23^ The processed Seurat object was subsetted to remove metastatic and non-melanoma cells, yielding only primary UM cells. Signature activity per cell was quantified using AddModuleScore(). Cells were classified as “high-scoring” if their module score exceeded the 75^th^ percentile of all primary tumor cells. Differences in the proportion of high-scoring cells between GEP Class 1 and Class 2 were assessed using the chi-squared test. Overall score distributions were compared using the Wilcoxon rank-sum test with rank-biserial correlation as a measure of effect size.

### Quantification and statistical analysis

All statistical analyses were performed in R (v4.4.0–4.4.1). Differentially expressed genes were identified using the Wilcoxon rank-sum test implemented in Seurat’s FindAllMarkers() function, with thresholds of log_2_FC > 2 and min.pct = 0.1 for melanocyte clustering, and log_2_ fold-change ≥0.5, delta percent expression (pct.1 - pct.2) ≥ 0.25, expressed in ≥20% of Cluster 2 cells (pct.1 ≥ 0.20), and expressed in ≤10% of other clusters (pct.2 ≤ 0.10) for the Cluster 2-specific 190-gene signature. Gene Ontology and KEGG enrichment analyses used Benjamini-Hochberg adjusted q < 0.05 as the significance threshold. GSEA was performed with the Benjamini-Hochberg correction (adjusted *p* < 0.05). Enrichment of Cluster 2 melanocytes by anatomical location and developmental age was assessed using Fisher’s exact test on 2 × 2 contingency tables.

Association between Cluster 2 gene signature scores and SCNA molecular risk class in TCGA-UVM as assessed using Spearman’s rank correlation and the Kruskal-Wallis test. Individual gene-level correlations with SCNA risk class were calculated using Spearman’s rank correlation. In independent microarray cohorts, association between signature scores and chromosome 3 status was assessed using Spearman’s rank correlation and the Wilcoxon rank-sum test. In the UM scRNA-seq dataset, differences in the proportion of high-scoring cells between GEP Class 1 and Class 2 were assessed using the chi-squared test and overall score distributions were compared using the Wilcoxon rank0sum test with rank-biserial correlation as a measure of effect size. Statistical significance was defined as *p* < 0.05 throughout.

## Supplemental information

Document S1. Figures S1–S5 and Tables S2, S4, and S5

Table S1. 114 genes with near-exclusive expression in melanocyte cluster 2, ranked by Δ-expression, related to Figure 4

Table S3. 190 gene list (cluster 2 signature) derived from log_2_FC ≥ 0.5, Δ% ≥ 0.25, ≥ 20% expression in cluster 2, and ≤ 10% expression in other melanocytes, related to Figure 10
