# Supplementary material for: Single-cell transcriptomic atlas of the human fetal uveal tract reveals heterogeneity in melanocyte populations
Source: iScience. 2026 Sep 12;29(10):117510. doi: 10.1016/j.isci.2026.117510 (PMC13590050; doi:10.1016/j.isci.2026.117510)
Supplement: Supplementary file 1 [file mmc1.pdf]

iScience, Volume *29*

Supplemental information

Single-cell transcriptomic atlas of the human fetal uveal tract reveals heterogeneity in melanocyte populations

James Draper, Ian R. Reekie, Lakshanie Wickramasinghe, Srilakshmi Sharma, Mark Coles, Andrew D. Dick, Christopher Buckley, Helen Kalirai, and Sarah E. Coupland

Supplementary Figures and Tables

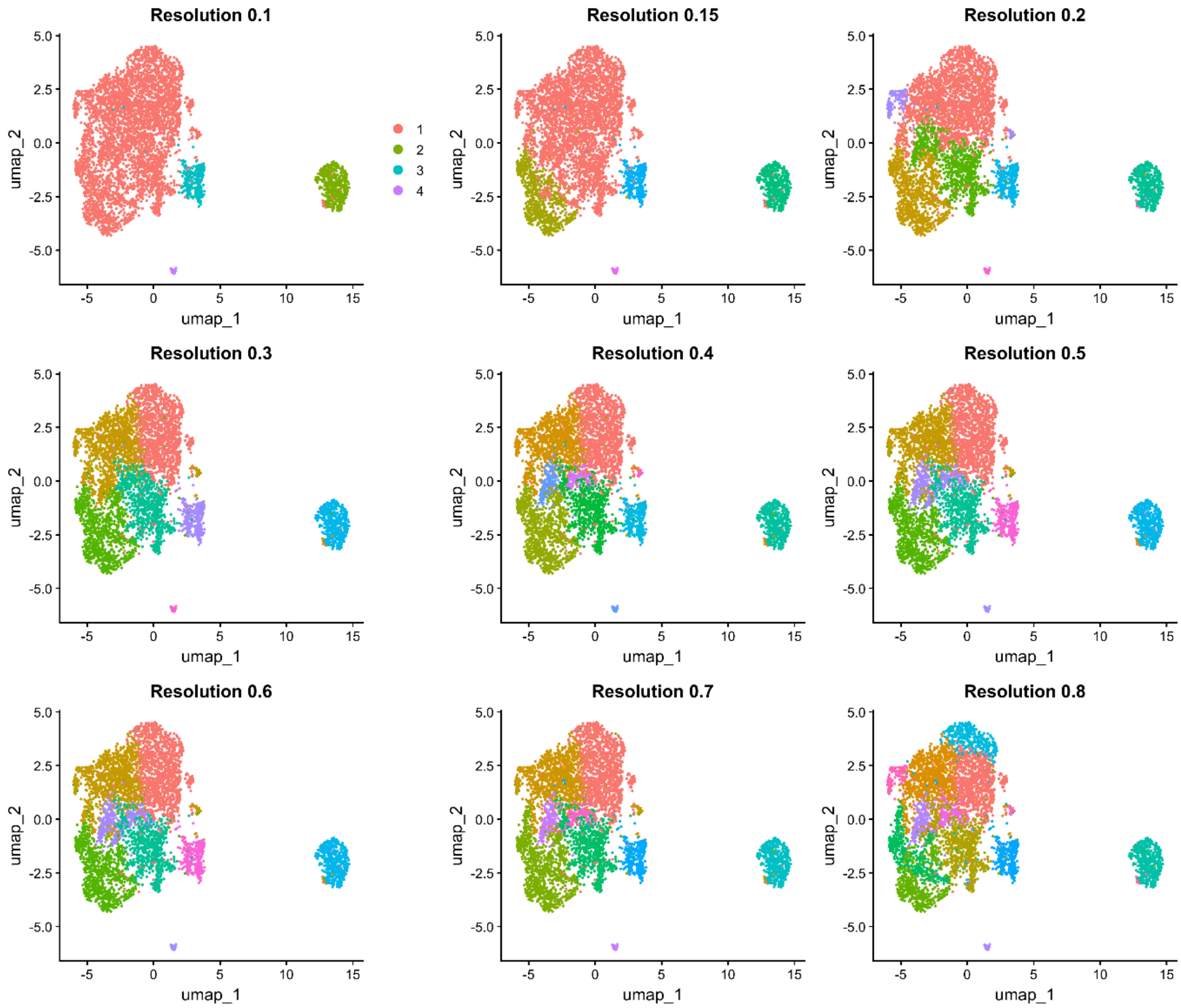

Supplementary Figure 1: Stability assessment of melanocyte clusters across tested resolutions (0.1-0.8), Leiden algorithm.

Cell populations accounting for clusters 2, 3 and 4 at resolution 0.1 remain stable and unfragmented throughout tested resolutions, implying a distinct transcriptional phenotype.

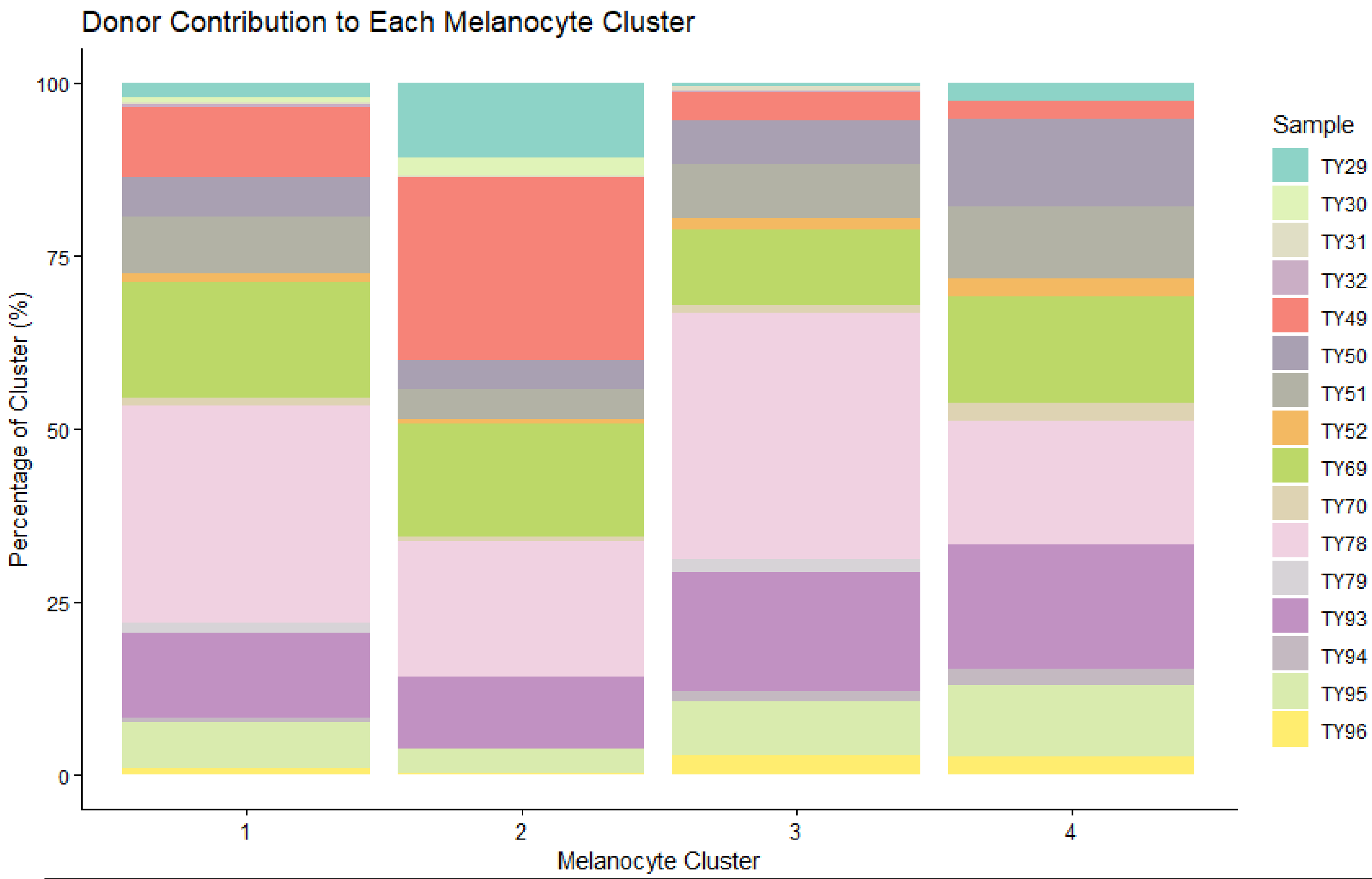

Supplementary Figure 2: Proportional contribution of all 16 original samples to each of the 4 melanocyte clusters.

Stacked bar chart showing the distribution of cells from each donor sample across the four melanocyte clusters. Each colour represents a unique donor sample

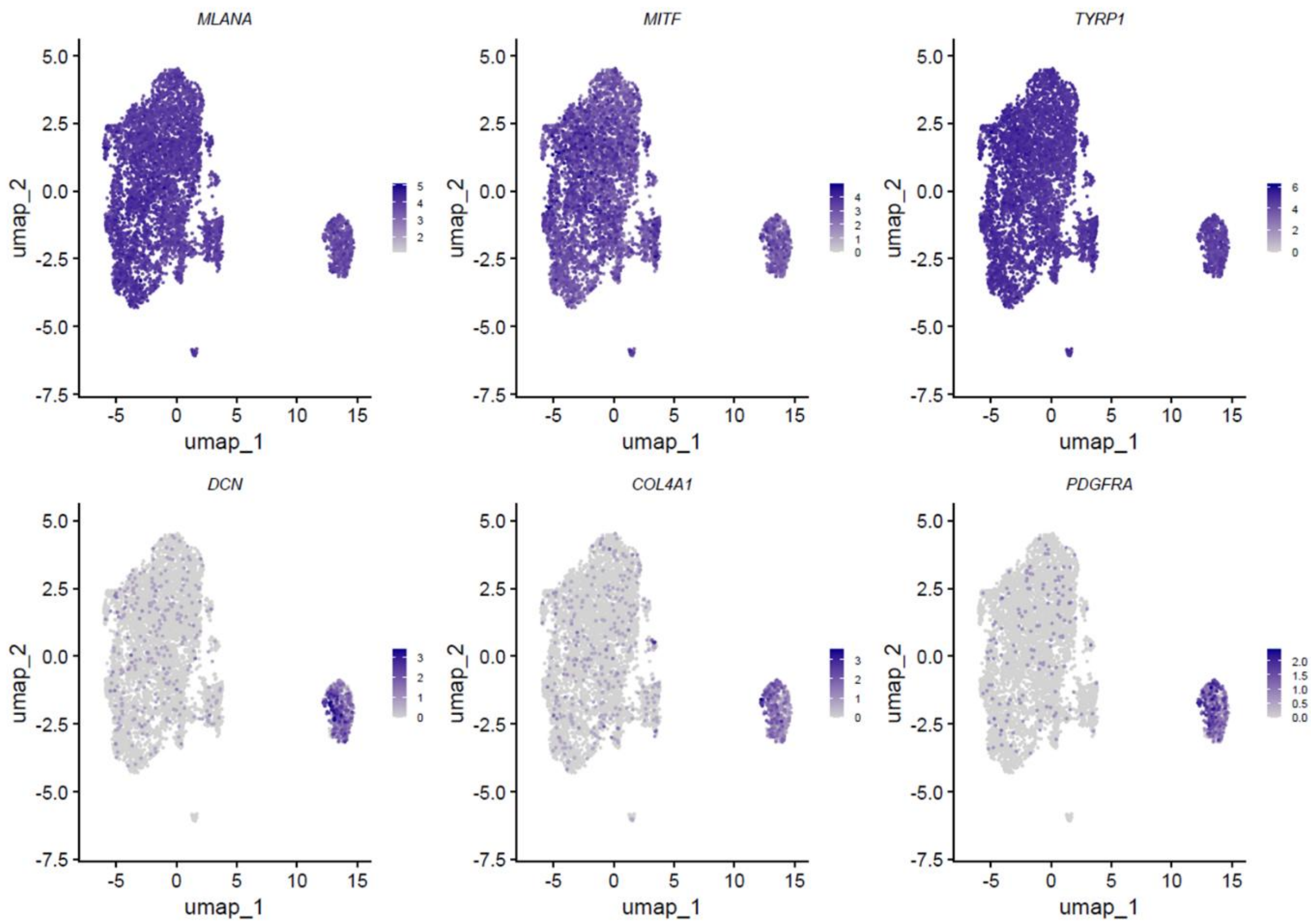

Supplementary Figure 3. Co-expression of canonical melanocyte and mesenchymal markers in cluster 2 melanocytes.

Feature plots showing normalised gene expression across the melanocyte UMAP for canonical melanocyte markers (MLANA, MITF, TYRP1, top row) and Cluster 2 enriched mesenchymal genes (DCN, COL4A1, PDGFRA, bottom row). Co-localisation of canonical melanocyte marker expression with mesenchymal gene expression in the cluster 2 region of the UMAP supports interpretation that this subpopulation represents a transcriptionally distinct melanocyte state rather than fibroblast contamination. Colour scale represents normalised gene expression; grey indicates undetected or low expression.

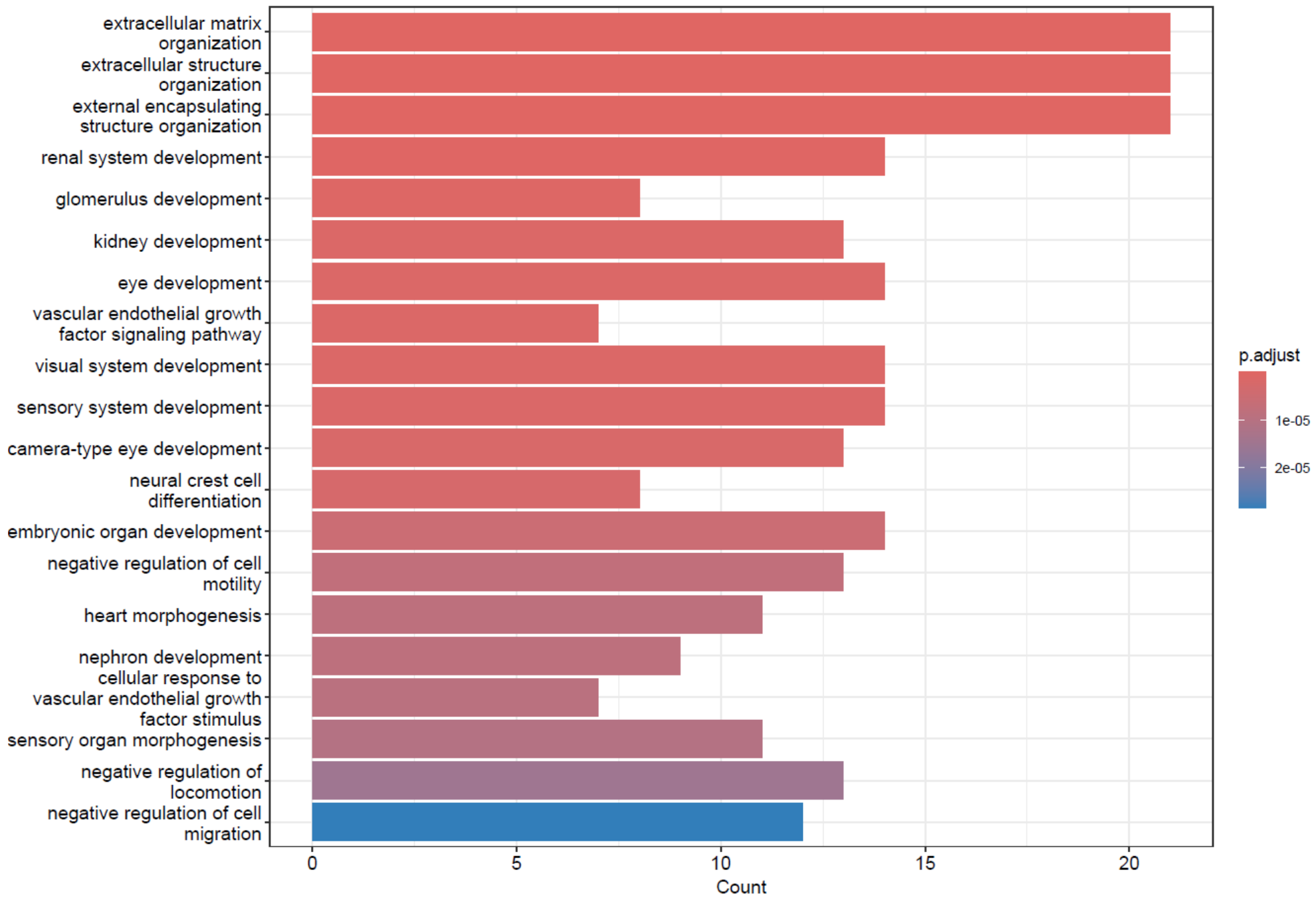

Supplementary Figure 4: GO BP bar plot of 190 gene Cluster 2 Melanocyte gene signature($\log_2 FC \geq 0.5$, Δ% ≥ 0.25, ≥ 20% expression in Cluster 2 & ≤10% expression in other melanocytes).

Upregulated processes associated with this gene signature include extracellular matrix processes, tissue morphogenesis and cellular differentiation.

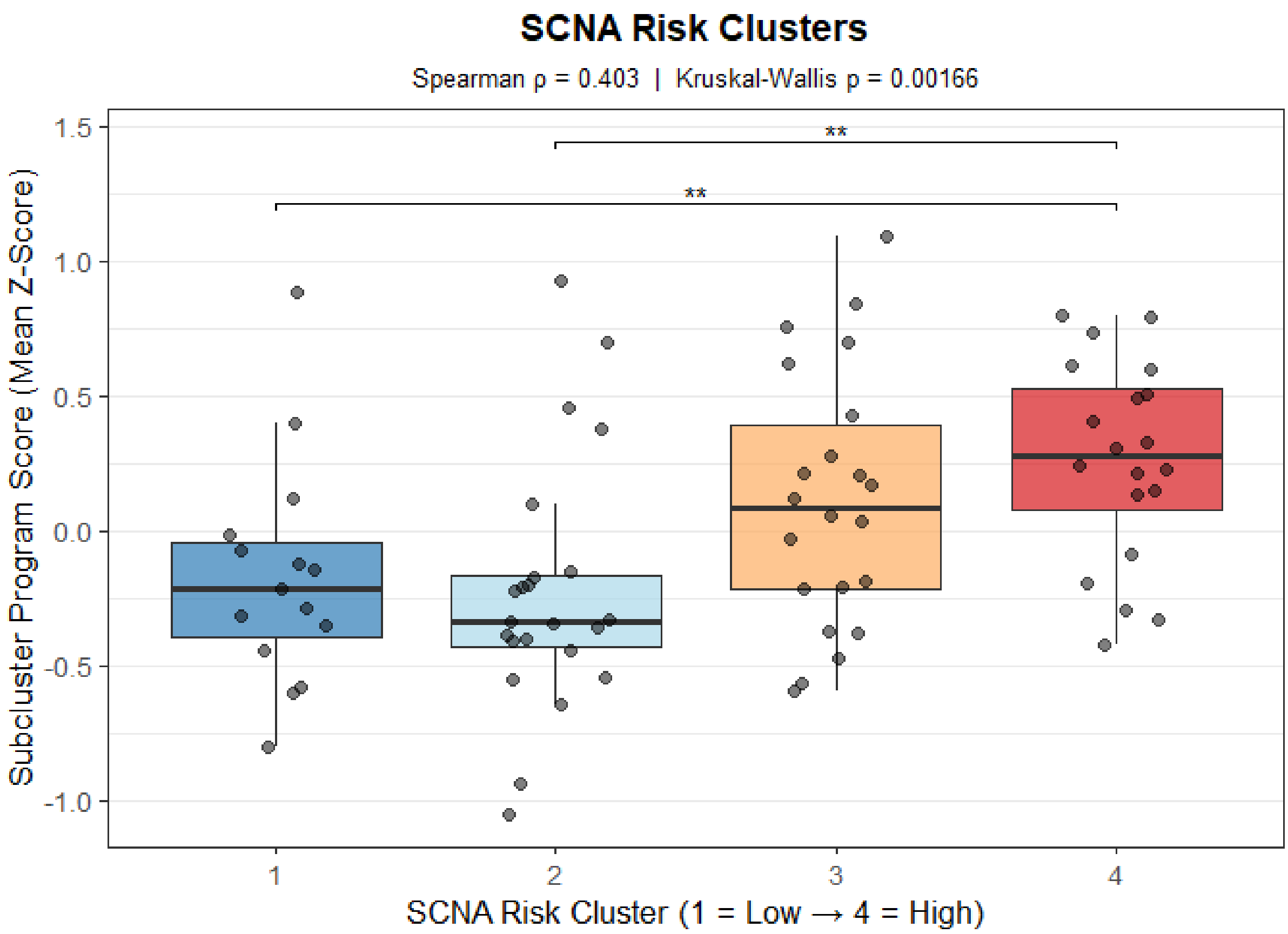

Supplementary Figure 5: Cluster 2 190-gene signature associated with SCNA-based molecular risk clusters in uveal melanoma.

Boxplots showing the distribution of cluster 2 gene signature scores across SCNA risk clusters (1 = low risk, 4 = high risk) in 80 TCGA-UVM samples. Boxes show median and interquartile range. Gene signature score was calculated as a mean of whole cluster 2 gene module (171 matched genes). Each dot represents one patient sample. Spearman $\rho = 0.407$, $p = 0.0002$; Kruskal-Wallis $p = 0.001$.

Supplementary Table 2: Mean quality control metrics across the four melanocyte clusters

Cluster 2 shows elevated UMI and gene counts consistent with transcriptional complexity, while doublet scores, mitochondrial percentages, and ribosomal percentages remain comparable to other clusters, consistent with genuine single-cell identities.

Cluster	Cell Count	Mean # UMIs	Mean # Genes	Mean mitochondrial %	Mean ribosomal %	Mean doublet score
1	4370	14935.7	3383.25	2.852574	34.64157	0.23029
2	450	18601.1	4112.81	2.660722	28.95576	0.24682
3	283	20697.8	4206.1	2.956828	33.00984	0.24551
4	39	17026.3	3893.59	2.822842	27.15741	0.24325

Supplementary Table 4: Refined 20-gene Cluster 2 melanocyte signature ranked by individual Spearman correlation with SCNA-based molecular risk class in TCGA-UVM (n=80).

Gene	Spearman ρ (TCGA-UVM)	p_value	p_adj
SHISA2	0.743663	2.75E-15	3.31E-13
HTRA1	0.741139	3.82E-15	3.31E-13
TGFBI	0.709936	1.67E-13	9.60E-12
SETBP1	0.65225	5.59E-11	2.42E-09
MRC2	0.620101	8.57E-10	2.96E-08
MAP1B	0.606125	2.56E-09	7.37E-08
SULF2	0.592756	6.92E-09	1.35E-07
COL12A1	0.59147	7.60E-09	1.35E-07
COL4A1	0.591082	7.82E-09	1.35E-07
COL14A1	0.589481	8.78E-09	1.38E-07
PTGS2	0.573728	2.65E-08	3.82E-07
TWIST1	0.558775	7.18E-08	9.56E-07
COL4A2	0.555961	8.62E-08	9.98E-07
FST	0.555895	8.65E-08	9.98E-07
RASL11A	0.554493	9.47E-08	1.02E-06
SEC24D	0.553	1.04E-07	1.06E-06
LPAR1	0.551169	1.17E-07	1.13E-06
FOXC1	0.543708	1.87E-07	1.62E-06
HIC1	0.535373	3.11E-07	2.56E-06
PRRX1	0.517127	9.05E-07	6.80E-06

Supplementary Table 5: External uveal melanoma datasets used for signature validation.

Dataset	Data Type	Platform	N Samples / Cells	Tumour Site	Risk Stratification	Risk Groups
TCGA-UVM	Bulk RNA-Seq	Illumina HiSeq	80 samples	Primary	SCNA risk cluster	Class 1: 15 Class 2: 23, Class 3: 22: Class 4: 20
GSE22138	Bulk microarray	Affymetrix U133 Plus 2.0	55 samples*	Primary	Chromosome 3 status	Disomy: 18, Monosomy: 37
GSE84976	Bulk microarray	Illumina HumanHT-12	28 samples	Primary	Chromsome 3 status	Disomy: 14, Monosomy: 14
GSE139829	scRNA-seq	10x Genomics	31,340 cells (8 tumours)	Primary**	GEP class	Class 1: 10,790 cells (2 tumours) Class 2: 20,550 cells, 6 tumours)

* Eight samples excluded due to missing chromosome 3 status annotation (original n=63).

** Metastatic data is contained within original dataset; metastatic cells were manually removed for this study.
